# Divergent methyl-coenzyme M reductase genes in a deep-subseafloor Archaeoglobi

**DOI:** 10.1038/s41396-018-0343-2

**Published:** 2019-01-16

**Authors:** Joel A. Boyd, Sean P. Jungbluth, Andy O. Leu, Paul N. Evans, Ben J. Woodcroft, Grayson L. Chadwick, Victoria J. Orphan, Jan P. Amend, Michael S. Rappé, Gene W. Tyson

**Affiliations:** 1 Australian Centre for Ecogenomics, School of Chemistry and Molecular Biosciences, University of Queensland, St Lucia, QLD Australia; 2 Center for Dark Energy Biosphere Investigations, University of Southern California, Los Angeles, CA USA; 3 Department of Energy, Joint Genome Institute, Walnut Creek, CA USA; 4 Division of Geological and Planetary Sciences, California Institute of Technology, Pasadena, CA USA; 5 Departments of Earth Sciences and Biological Sciences, University of Southern California, Los Angeles, CA USA; 6 Hawaii Institute of Marine Biology, University of Hawaii at Manoa, Kaneohe, HI USA

**Keywords:** Archaeal genomics, Metagenomics, Microbial ecology

## Abstract

The methyl-coenzyme M reductase (MCR) complex is a key enzyme in archaeal methane generation and has recently been proposed to also be involved in the oxidation of short-chain hydrocarbons including methane, butane, and potentially propane. The number of archaeal clades encoding the MCR continues to grow, suggesting that this complex was inherited from an ancient ancestor, or has undergone extensive horizontal gene transfer. Expanding the representation of MCR-encoding lineages through metagenomic approaches will help resolve the evolutionary history of this complex. Here, a near-complete Archaeoglobi metagenome-assembled genome (MAG; *Ca*. Polytropus marinifundus gen. nov. sp. nov.) was recovered from the deep subseafloor along the Juan de Fuca Ridge flank that encodes two divergent McrABG operons similar to those found in *Ca*. Bathyarchaeota and *Ca*. Syntrophoarchaeum MAGs. *Ca*. P. marinifundus is basal to members of the class Archaeoglobi, and encodes the genes for β-oxidation, potentially allowing an alkanotrophic metabolism similar to that proposed for *Ca*. Syntrophoarchaeum. *Ca*. P. marinifundus also encodes a respiratory electron transport chain that can potentially utilize nitrate, iron, and sulfur compounds as electron acceptors. Phylogenetic analysis suggests that the *Ca*. P. marinifundus MCR operons were horizontally transferred, changing our understanding of the evolution and distribution of this complex in the Archaea.

## Introduction

The methyl-coenzyme M reductase (MCR) complex is a key component of methane metabolism, and until recently had only been found within the Euryarchaeota (*Methanococcales, Methanopyrales, Methanobacteriales, Methanomicrobiales, Methanocellales, Methanosarcinales, Methanomassiliicoccales, Methanofastiosales, Methanoflorentales, Methanophagales* [1] [ANME-1], and *Methanonatronarchaeia*). However, recent genome-centric metagenomic studies have led to the discovery of genomes encoding MCR complexes within the phylum *Candidatus* Bathyarchaeota and the crenarchaeal family *Candidatus* Methanomethyliaceae (previously Verstraetearchaeota) [2, 3]. Originally, the novel MCR-encoding *Ca*. Bathyarchaeota and *Ca*. Methanomethyliaceae were inferred to be capable of hydrogenotrophic and methylotrophic methanogenesis, respectively. Intriguingly, the *Ca*. Bathyarchaeota also appeared to be capable of producing energy through peptide fermentation and β-oxidation, unusual among MCR-encoding microorganisms. More recently a euryarchaeotal lineage, *Candidatus* Syntrophoarchaeum, was found to encode *Ca*. Bathyarchaeota-like MCR homologs and experimentally demonstrated to activate butane for oxidation via modified β-oxidation and Wood-Ljungdahl (WL) pathways [4]. The similarity in the MCR complexes and inferred metabolism of the *Ca*. Bathyarchaeota and *Ca*. Syntrophoarchaeum suggest that the *Ca*. Bathyarchaeota may also oxidize short hydrocarbons. Both organisms are confined to anoxic, hydrocarbon-rich habitats [2–4], where abiotically produced short alkanes are abundant and likely to be utilized as carbon and energy sources. The increased number of archaeal lineages encoding the MCR complex and their metabolic flexibility suggests that these microorganisms may have a greater impact on carbon cycling than originally suspected.

The similarity of the MCR complexes encoded by *Ca*. Bathyarchaeota and *Ca*. Syntrophoarchaeum is incongruent with their large phylogenetic distance in the genome tree, suggesting that these genes were acquired via horizontal gene transfer (HGT) [1, 5]. Both scenarios indicate that further diversity of divergent MCR-encoding lineages remains to be discovered [5], which has been supported by gene-centric metagenomic analyses of deep-sea and terrestrial hydrothermal environments [6, 7]. Expanding the genomic representation of novel MCR-encoding lineages by targeting these environments using genome-centric metagenomic approaches will help resolve the evolutionary history of the complex and expand the diversity of lineages known to be involved in hydrocarbon cycling.

Archaeoglobi is a class of thermophilic Archaea belonging to the Euryarchaeota that are abundant in subsurface hydrothermal environments, where they likely play a role in carbon and nutrient cycling [8, 9]. The Archaeoglobi are split into three genera: *Archaeoglobus*, which are all heterotrophic or chemolithotrophic sulfate reducers [10–19], and *Geoglobus* and *Ferroglobus*, which reduce both nitrate and ferric iron [20–22]. Pure cultures of *Archaeoglobus* have been shown to be capable of alkane oxidation [23, 24], and based on their shared metabolic features and close phylogenetic relationship with methanogens [25–28] are suggested to have an ancestor capable of methanogenesis. However, there are currently no representatives of the Archaeoglobi known to encode the MCR complex, likely a result of poor genomic representation caused by their extreme habitats that are difficult to sample.

Borehole observatories installed on the flank of the Juan de Fuca Ridge in the Pacific Ocean provide pristine fluids from the subseafloor igneous basement aquifer [29]. Previous metagenomic studies on samples collected from these borehole observatories revealed a distinct microbial community, including a number of novel Archaeoglobi [30, 31]. Here, we characterize metagenome assembled genomes (MAGs) from igneous basement fluid samples from the boreholes [31], focusing on a genome encoding two divergent copies of the *mcrABG* operon. Metabolic reconstruction revealed that the novel Archaeoglobi is potentially capable of hydrocarbon oxidation, amino acid fermentation, and can utilize multiple electron acceptors. Phylogenetic analyses support a horizontal gene transfer hypothesis for the distribution of novel MCR complex among the Archaea, and provides insight into the evolution of both the MCR complex and the Archaeoglobi.

## Materials and methods

### Metagenome generation, assembly, and binning

Two metagenomes from crustal fluids of the JdFR flank were generated as described previously [31]. One sample (SRR3723048) that yielded a novel Archaeoglobi genome was selected for reassembly using metaSPAdes v3.9.0 [32] with default settings, using raw reads as input. Raw reads from SRR3723048 were mapped to the resulting assembly using BWA-MEM [33] v0.7.12. Binning was conducted using MetaBAT v0.32.4 [34] using the --specific setting.

### Identification of MCR encoding genomes

Genomes generated by MetaBAT were searched with GraftM v0.11.1 [6] using an McrA-specific GraftM package (gpkg). The McrA gene tree was curated with NCBI taxonomy, with the Bathyarchaeota and Syntrophoarcaheum clade labeled as “divergent”. An evalue of 1e-50 was used to filter for full-length McrA genes.

### MAG quality control

The genome encoding the divergent MCR complexes was analysed using RefineM [35] v0.0.23 to identify contigs with divergent tetranucleotide frequencies and GC content. A single 2748 bp contig was removed due to divergent a GC, tetranucleotide and taxon profile (Supplementary Note 1). The remaining contigs were scaffolded with FinishM v0.0.7 roundup using default parameters (github.com/wwood/finishm). The completeness and contamination of the resulting bin was assessed using CheckM v1.0.8 [36] with default settings.

### Genome annotation

Genomes were annotated using EnrichM annotate (github.com/geronimp/enrichM). Briefly, EnrichM calls proteins from contigs using Prodigal v2.6.3 [37], and blasts them against UniRef100 using DIAMOND [38] v0.9.22 to obtain KO annotations. Pfam-A [39] (release 32) and TIGRFAM [40] (release 15.0) Hidden Markov Models (HMMs) were run on the proteins using hmmer 3.1b [41] to obtain Pfam and TIGRFAM annotations, respectively. Further manual curation was completed using NCBI BLAST and CD-Search [42].

### Genome tree

Using GenomeTreeTK (https://github.com/dparks1134/GenomeTreeTk) v0.0.41, a genome tree of Archaea from NCBI’s RefSeq database (release 80) was created using a concatenated alignment of 122 archaea-specific single marker copy genes. Genomes <50% complete, and with >10% contamination as determined using CheckM were removed from the analysis. After alignment to HMMs constructed for each of the 122 marker genes, alignments were concatenated and genomes with <50% of the alignment were excluded from the analysis. Maximum likelihood trees were constructed using FastTree v2.1.9, and non-parametric bootstrapping was completed using GenomeTreeTK’s bootstrap function.

### 16S rRNA gene tree

Sequences classified as Archaeoglobi with a pintail score of 100 and an alignment and sequence quality of ≥80 were extracted from the SILVA database [43] (version 132) and used as reference sequences for a 16S rRNA phylogenetic tree. The partial 16S rRNA sequence from the genome encoding the novel MCR complexes was added to the database, sequences were aligned using ssu-align [44] v0.1, and subsequently converted to fasta format using the convert mode from seqmagick v0.6.1 (fhcrc.github.io/seqmagick). Gapped regions in the alignment were removed with trimAl v1.2 using the --gappyout flag [45]. The Maximum likelihood tree was constructed using FastTreeMP [46] with a generalized time-reversible model and --nt flags, bootstrapped with GenomeTreeTK (github.com/dparks1134/GenomeTreeTk), and visualized in ARB [47] v6.0.6.

### Protein phylogenies (McrA, McrB, McrG, and RuBisCo)

McrA, McrB, McrG, and RuBisCo sequences were derived from the genomes used in the genome tree. Proteins from all genomes were called using Prodigal v2.6.3, and searched using hmmer v3.1b with Pfam Hidden Markov models (PF02240.15, MCR_gamma; PF02241.17, MCR_beta; PF02249.16, MCR_alpha; PF02745.14, MCR_alpha_N; PF00016.19, RuBisCO_large; PF02788.15, RuBisCO_large_N) with the –cut_tc flag to minimize false positives. For McrA and RuBisCo, both models needed to hit a sequence for it to be included in the analysis. For each gene, sequences were aligned using MAFFT-GINS-i v7.221 and filtered using trimAl with the --gappyout flag. A maximum likelihood tree was constructed using FastTreeMP with default parameters.

### Average amino acid identity

Average amino acid identity was calculated with CompareM v0.0.22 (https://github.com/dparks1134/CompareM) using the aai_wf with default parameters.

### Network analysis of MHCs

Proteins from the Archaea in NCBI’s RefSeq database (release 80) were searched with the Cytochrome C Pfam HMM (PF00034). Hits were filtered to have at least one of the characteristic cytochrome C CXXCH domains using a custom script (fastacxxch.count.py, github.com/geronimp/HandyScripts/blob/master/99_random/fastacxxch.count.py). After removing duplicate sequences, the closest match for all resulting proteins were identified using DIAMOND with an evalue cutoff of 1e−20. No limit was placed on the number of hits for each protein. The result was visualized in Cytoscape v3.2.0, removing clusters without a *Ca*. P. marinifundus homolog.

### KO analysis

Proteins from the genomes were searched using DIAMOND blastp against UniRef100 with an evalue cutoff of 1e-05. For each protein, the KO annotations were derived from the top hit. The presence/absence of each KO in each genome was used as input to a principal component analysis (PCA) using the prcomp function in R.

### Sliding window GC and tetranucleotide frequencies

A custom script (https://github.com/geronimp/window_sequence) was written to fragment contigs into short sequences, in a sliding window. For each fragment, the percent GC and 4mer frequency was calculated using seqstat in the biosquid package v1.9 g (packages.debian.org/sid/biosquid) and Jellyfish [48] v2.2.6.

### Data visualization

Figures were generated in R [49] v3.0.1 using ggplot [50] v1.0.0 and refined using Inkscape v0.91 (inkscape.org/en).

## Results and discussion

To investigate the microbial diversity within Juan de Fuca Ridge flank boreholes, a metagenome (45.4 Gbp total raw reads) was generated, assembled, and binned. One of the 98 MAGs (Supplementary Note 1; Supplementary Figure 1) was found to encode two divergent McrAs that were most similar to *Ca*. Syntrophoarchaeum caldarius (52% AAI) and *Ca*. Syntrophoarchaeum butanivorans (56% AAI). Based on 228 Euryarchaeota-specific marker genes, this MAG was estimated to be nearly complete (99.84%) with low contamination (1.96%), and a genome size of ~2.13 Mbp. Annotation of the 2305 proteins this MAG encoded revealed all subunits of the MCR complex arranged in operons (*mcrABG*), including two copies of the *mcrC* subunit and an ancillary *mcrD* subunit, all of which have highest sequence similarity to homologs within *Ca*. Syntrophoarchaeum.

**Fig. 1:**
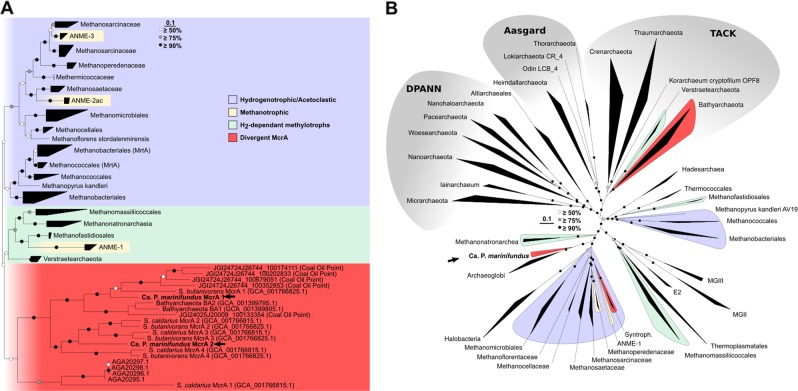
McrA gene phylogeny and genome tree phylogeny. Maximum-likelihood trees of **a** McrA proteins, and **b** a concatenated alignment of 122 single copy archaeal marker genes from high quality archaeal RefSeq genomes (release 80). Hydrogenotrophic and acetoclastic methanogens are shaded blue, H_2_-dependent methylotrophic methanogens are shaded green, known/putative methane oxidizers are shaded yellow, and lineages encoding divergent McrAs are shaded red. Bootstrap support was generated from 100 replicates, and white, gray and black nodes represent ≥50%, ≥75%, and ≥90% support, respectively

Phylogenetic analysis of the two MCR complexes encoded by the MAG revealed that they branched with high support with divergent MCRs from *Ca*. Syntrophoarchaeum and *Ca*. Bathyarchaeota (Fig. 1a; Supplementary Figure 2–6). Notably, the average branch length within the divergent McrA clade was double (1.05 ± 0.24 substitutions per site) that of traditional hydrogenotrophic, acetoclastic and H_2_-dependent methylotrophic methanogens (0.46 ± 0.10 substitutions per site), suggesting an accelerated rate of evolution following duplication or HGT [51], the latter being more likely due the fewer evolutionary events required. To determine the taxonomy of the MAG, a genome tree was constructed from a concatenated alignment of 122 archaeal single copy marker genes. In both FastTree and IQ-TREE phylogenies, the MAG was positioned basal to other members within the class Archaeoglobi with strong bootstrap support (100%; Fig. 1b; Supplementary Figure 7,8), including other Archaeoglobi previously recovered from the Juan de Fuca Ridge [31]. Phylogenetic analysis of the partial 16S rRNA gene (904 bp) confirmed the position of the MAG within the Archaeoglobi (Supplementary Figure 9). The average AAI between the MAG and other Archaeoglobi recovered from the Juan de Fuca Ridge (54.2% ± 0.7 AAI; Supplementary Figure 10) and relative evolutionary divergence [52, 53], suggest it is the first representative of a novel family within the Archaeoglobi [54]. We propose the name *Candidatus* ‘Polytropus marinifundus’ gen. nov., sp. nov. for this MAG, as the first representative of a new family within the Archaeoglobi, *Candidatus* ‘Polytropaceae fam. nov. The incongruencies between the genome tree and MCR phylogenies for *Ca*. P. marinifundus, *Ca*. Syntrophoarchaeum, and the *Ca*. Bathyarchaeota are most parsimoniously explained by HGT of the MCR.

**Fig. 2:**
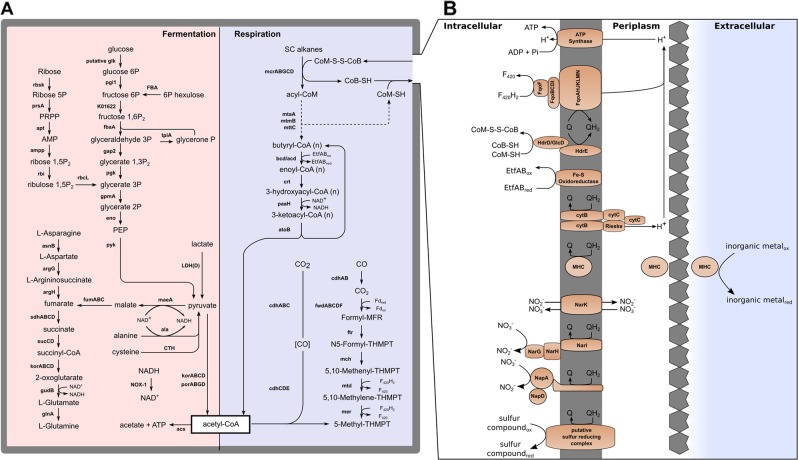
Metabolic reconstruction of the Ca. P. marinifundus genome. Metabolic reconstruction of the *Ca*. P. marinifundus genome. **a** Respiratory and fermentative pathways are shaded blue and red, respectively. **b** Proposed CoM-S-S-CoB disulfide regeneration and membrane energetics of *Ca*. P. marinifundus are shown. Genes associated with the pathways shown can be found in Supplementary Table 1

Metabolic reconstruction of the *Ca*. P. marinifundus MAG highlighted the potential for diverse metabolic capabilities, including amino acid fermentation and short chain alkane oxidation using a wide variety of electron acceptors (Fig. 2). *Ca*. P. marinifundus encodes a complete Wood-Ljungdahl pathway, and consistent with the *Ca*. Bathyarchaeota and *Ca*. Methanomethyliaceae, encodes five copies of the H subunit of the methyltetrahydromethanopterin (H_4_MPT): coenzyme M methyltransferase complex (*mtrH*), each co-located with predicted di-methylamine and tri-methylamine corrinoid proteins (Supplementary Table 1). This suggests that *Ca*. P. marinifundus encodes a diverse range of methyltransferases, but is unlikely to conserve energy via methane oxidation or hydrogenotrophic methanogenesis [26]. The WL pathway may be used for oxidation of acetyl-CoA as previously observed in heterotrophic Archaeoglobales isolates [12]. *Ca*. Syntrophoarchaeum caldarius and *Ca*. Syntrophoarchaeum butanivorans have been inferred to oxidize alkanes activated by the MCR complex, putatively via the β-oxidation and WL pathways [4]. Ca. P. marinifundus encodes β-oxidation and methyltransferase enzymes that would allow short alkane oxidation (Fig. 2), and the two copies of *mcrA* share catalytic residues with Ca. Syntrophoarchaeum homologs (Supplementary Figure 11). However, unlike *Ca*. Syntrophoarchaeum, the presence of a short chain acyl-CoA and butyryl-CoA dehydrogenase (*acd* and *bcd*, respectively), and a long-chain acyl-CoA synthetase (*fadD*) may allow *Ca*. P. marinifundus to oxidize long chain fatty acids (Fig. 2). The energy for hydrocarbon activation may be produced via either a soluble or membrane bound heterodisulfide reductase (*hdrABC*, *hdrDE*, respectively), both of which are encoded by the *Ca*. P. marinifundus genome. While *mvhAG* was not encoded by *Ca*. P. marinifundus, the C-terminal of *hdrA* is fused to the *mvhD* subunit as previously observed in *Methanosarcina acetivorans* [55], suggesting it plays a similar role in disulfide reduction. A further seven putative *hdrD* subunits co-located with flavin adenine dinucleotide-containing dehydrogenases (*glcD*) potentially oxidize coenzyme M (CoM-SH) and coenzyme B (CoB-SH) as proposed for the *Ca*. Methanomethyliaceae and *Ca*. Bathyarchaeota (Fig. 2) [2, 3]. While common in the Archaeoglobi, the membrane bound HdrDE has not been observed in *Ca*. Bathyarchaeota and *Ca*. Syntrophoarchaeum. The functional redundancy of *hdrABC*/*DE* has also been observed in *Archaeoglobus profundus* [56], where they were suggested to play a role in sulfur metabolism. However, without the dissimilatory sulfate reductase (*dsrAB*) gene, their role in *Ca*. P. marinifundus remains unclear.

**Fig. 3:**
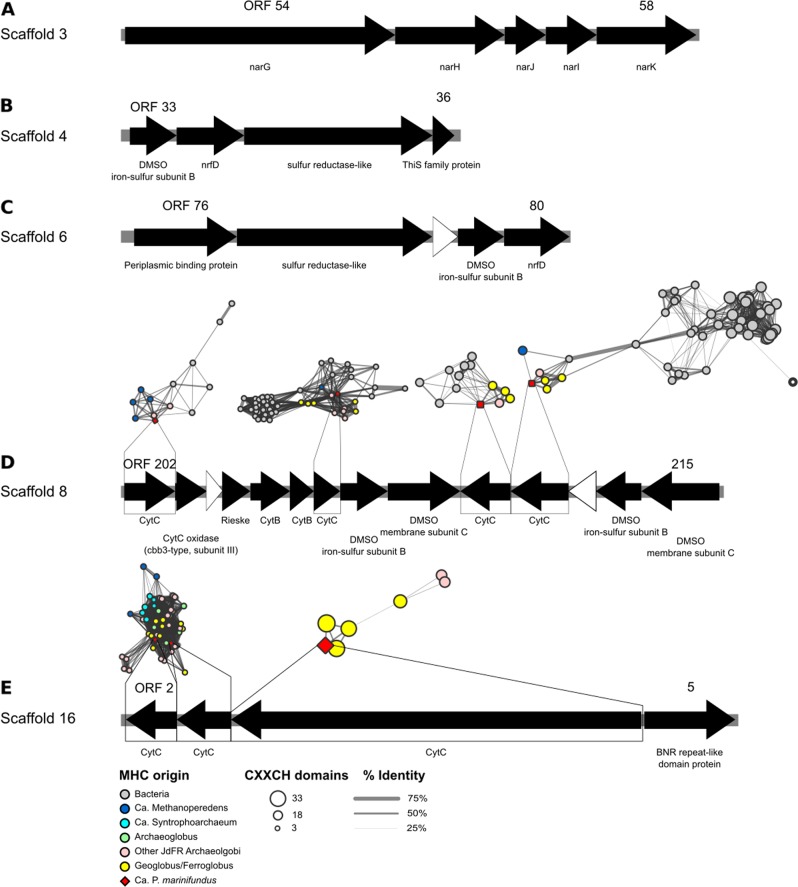
Operons encoding redox active complexes within *Ca*. P. marinifundus. White arrows represent hypothetical proteins. Networks are of MHCs and represent clusters of related proteins and their organism of origin

Alkane oxidation can be energetically favorable when coupled to an electron accepting process such as sulfate [57], nitrate [58], nitrite [59], and metal oxide [60] reduction, or transfer to a syntrophic partner via direct interspecies electron transfer (DIET) [61]. Similar to the iron metabolizing *Geoglobus* [22, 62, 63] and *Ferroglobus* [64] within the Archaeoglobi, *Ca*. P. marinifundus does not encode *dsrAB*, but was found to encode 10 multi-haem c-type cytochromes (MHCs) with 4–31 haem binding motifs that may facilitate iron reduction [8, 62, 63, 65] or DIET as proposed in ANME-2 [61]. To compare the multi-haem cytochrome profile of *Ca*. P. marinifundus with other Bacteria and Archaea, a network analysis was conducted on genomes from NCBI’s RefSeq database. Each MHC encoded by *Ca*. P. marinifundus share high sequence similarity to homologs encoded by archaeal (e.g., *Ferroglobus placidus*, *Geoglobus acetivorans*) and bacterial (e.g., *Ferrimonas* and *Shewanella*) iron reducers, *Ca*. Methanoperedens nitroreducens, and *Ca*. Syntrophoarchaeum (Fig. 3). Four multi-haem cytochromes similar to *Methanoperedens*, *Ferroglobus* and *Geoglobus* homologs are organized into three contiguous operons (Fig. 3d) encoding membrane-bound, redox-active complexes, including a *bc1*-like complex with a Rieske iron sulfur protein and cytochrome b that may generate a proton gradient with a Q-cycle, and two complexes associated with the transfer of electrons to the membrane (Fig. 3d). One complex includes an enzyme with two haem binding domains that is conserved among iron metabolizing *Geoglobus* and *Ferroglobus* (**ORF 212**). Intriguingly, *Ca*. P. marinifundus encodes an operon of three MHCs, two of which are specific to Archaeoglobi, *Methanoperedenaceae*, and *Ca*. Syntrophoarchaeum, suggesting these MHCs play a specific role in alkane oxidizers (Fig. 3e). The final gene in this operon is homologous to a large C-type cytochrome also found in *Geoglobus acetivorans* SBH6^T^ that is a possible genomic determinant of iron reduction [21]. *Ca*. P. marinifundus also encodes a dissimilatory nitrate reductase (*narGHJIK*; Fig. 3a), which may allow alkane oxidation coupled to nitrate reduction [58]. A further two operons encode sulfur reductase-like complexes that were previously only found in the hyperthermophile *Aquifex aeolicus* [66], and shown to allow tetrathionate, polysulfide, and elemental sulfur to be used as terminal electron acceptors (Fig. 3b, c). The potential to use partially oxidized forms of sulfur, nitrogen, and iron as electron acceptors suggests that *Ca*. P. marinifundus can use different electron sinks depending on environmental conditions [67].

*Ca*. P. marinifundus putatively generates ATP via anaerobic respiration of short chain alkanes. A proton motive force is generated by the F_420_H_2_:quinone oxidoreductase, and ATP is generated by an archaeal type ATP synthase (Fig. 2B). However, *Ca*. P. marinifundus is predicted to be a facultative fermenter, with the ability to ferment via a number of pathways (Fig. 2). While no glucose transporters could be identified, glucose can be fermented via glycolysis, producing acetyl-CoA via pyruvate-ferredoxin oxidoreductase (*por*) or 2-oxoglutarate ferredoxin oxidoreductase (*kor*). *Ca*. P. marinifundus also encodes genes to ferment organic acids such as lactate via lactate dehydrogenase (*ldh*) and succinate, fumarate and malate via a partial citric acid cycle (Fig. 2). A type II/III ribulose 1,5-bisphosphate carboxylase/oxygenase homologous to *Ca*. Bathyarchaeota, *Ca*. Methanomethyliaceae, and Altiarchaeales may play a role in nucleotide catabolism [2, 68] (Archaeal type RuBisCO; Supplementary Figure 8 and 9). The RuBisCo encoded by *Ca*. P. marinifundus contained all catalytic residues but one (K177 [69]), a feature shared with the subsurface dwelling *Altiarchaeales* (Supplementary File 16). A number of amino acid transporters and peptidases (tetrahedral aminopeptidase, peptidase family M50, Xaa-Pro dipeptidase, methionine aminopeptidase) indicate that *Ca*. P. marinifundus can also ferment peptides (Fig. 2). Pathways for the fermentation of glutamate, glutamine, alanine, cysteine, aspartate and asparagine exist, as well as a number of aminotransferases (*aspB*, *hisC*, *cobD*) and two copies of *por*, which is involved in the fermentation of aromatic amino acids in other hyperthermophiles [70]. *Ca*. P. marinifundus also encodes a benzoyl-CoA reductase complex (*bcrABCD*) indicating the potential to degrade aromatic compounds. The acetyl-CoA generated via glycolysis and via fermentation of organic and amino acids may be used via a reversed Wood-Ljungdahl pathway, or substrate level-phosphorylation using acetyl-CoA synthetase (*acs*) or acetate-CoA ligase (*acd*). The various fermentative strategies used by *Ca*. P. marinifundus suggest that it is adapted to a fluctuating availability of organic compounds.

**Fig. 4:**
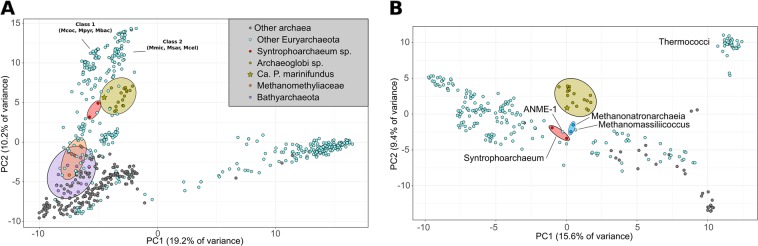
Comparative genomics of the *Ca*. P. marinifundus genome. **a** PCA of the presence/absence of KEGG Orthologous (KO) genes in all archaea, **b** within the Euryarchaeota, with the exception of the Haloarchaea, and **c** within the Archaeoglobi

To compare the metabolic capabilities of *Ca*. P. marinifundus with publically available archaeal genomes from RefSeq and GenBank, a global analyses of KEGG Orthologous (KO) genes was conducted. The KO profile of *Ca*. P. marinifundus was most similar to other members of the Archaeoglobi, *Methanomassiliicoccales*, *Methanophagales* [1] (ANME-1), *Ca*. Syntrophoarchaeales and *Methanonatronarchaeia sp*., but distant from the *Ca*. Bathyarchaeota and *Ca*. Methanomethyliaceae (Fig. 4a, b). To further examine the shared genomic content of novel MCR encoding lineages, orthologous clusters (OCs) were generated using proteinortho [71]. Within the Archaeoglobi, 134 OCs were unique to *Ca*. P. marinifundus, mapping to 71 KOs primarily associated with carbon metabolism (Supplementary Table 1). The few clusters that were unique to the *Ca*. P. marinifundus, *Ca*. Bathyarchaeota and *Ca*. Syntrophoarchaeum (seven OCs) were limited to subunits from the MCR complex, a putative methanogenesis marker (TIGR03275), and a gene associated with cobalamin biosynthesis (cob(I)alamin adenosyltransferase; *cobA*; Supplementary Table 1). A further 25 OCs were specific only to *Ca*. P. marinifundus and *Ca*. Syntrophoarchaeum, including four further methanogenesis markers (TIGR03271, TIGR03291, TIGR03268, TIGR03282), a sugar-specific transcriptional regulator, and a class II fumarate hydratase. Many OCs shared between *Ca*. P. marinifundus and *Ca*. Syntrophoarchaeum were annotated as “hypothetical protein”, suggesting much of the metabolic similarities of novel hydrocarbon metabolisers have yet to be functionally characterized.

To explore the evolutionary history of the MCR complex, the gene phylogeny of the McrA subunit was compared with the archaeal genome phylogeny (Supplementary Figure 14). Traditional euryarchaeal methanogens are largely congruent with the branching order of the genome tree (Supplementary Figure 14), suggesting that the evolutionary history of the McrA largely follows vertical inheritance. However, the H_2_-dependent methylotroph and *Ca*. Bathyarchaeota/*Ca*. Syntrophoarchaeum/*Ca*. P. marinifundus clades are not monophyletic in the genome tree (Supplementary Figure 14). *Ca*. Bathyarchaeota and *Ca*. P. marinifundus McrA cluster with different homologs of the *Ca*. Syntrophoarchaeum (Fig. 1a), a phylogenetic pattern most parsimoniously explained by HGT (Supplementary Figure 14). The basal phylogenetic position of *Ca*. P. marinifundus and divergence to other *Ca*. Syntrophoarcheum MCRcrA would suggest either the donor is unknown or this event did not occur in recent evolutionary history, supported by the lack of a divergent GC or kmer profile surrounding the gene context of the MCRs encoded by *Ca*. P. marinifundus [72] (Supplementary Figure 15 and 16). Potential HGT to an ancestor of *Ca*. P. marinifundus contradicts the traditional vertical inheritance pattern of the MCR complex within the Archaea. Syntrophoarchaeum and *Ca*. P. marinifundus encode multiple copies of the *mcrABG* operon, suggesting they may be expressed under different environmental conditions as observed for the *mcr* and *mrt* of the *Methanococci* and *Methanobacteria* [73], or are used for the oxidation of different length alkanes, as is the hypothesized for the *Ca*. Syntrophoarchaeum [4].

Given the metabolic similarities between the Archaeoglobi and methanogens [26], it has been hypothesized that the last common ancestor (LCA) of the Archaeoglobi encoded the MCR complex, which was subsequently lost following HGT of the *dsrAB* gene from the Bacteria [74, 75]. While it is unclear whether the LCA of the Archaeoglobi encoded a conventional or divergent MCR complex, four scenarios explain the current distribution of *dsrAB* and the MCR complex in this lineage: (i) HGT of *dsrAB* into the LCA of the Archaeoglobi, followed by loss of this metabolism after acquisition of the divergent MCR complex in *Ca*. P. marinifundus (Supplementary Figure 17A), (ii) HGT of the divergent MCR complex into the LCA of the Archaeoglobi, followed by loss of the complex after the acquisition of *dsrAB* in the Archaeoglobaceae (Supplementary Figure 17B) (iii) the separate acquisition of the divergent MCR complex and *dsrAB* by *Ca*. P. marinifundus and the Archaeoglobaceae (Supplementary Figure 17C), or (iv) differential loss of the *dsrAB* gene and the divergent MCR complex from an ancestor to the Archaeoglobi that encoded both operons (Supplementary Figure 17D). Greater genomic representation and comparative genomics of the Archaeoglobi will clarify the evolutionary story, particularly if lineages are found within the Archaeoglobi that encode the MCR complex. The evolutionary history of the MCR is highly complex, with evidence for both vertical inheritance and HGT (Supplementary Figure 17). It is likely that archaeal lineages encoding the divergent MCR complex will continue to be discovered, and with the genomic representation they add to public databases, their evolutionary history and metabolic role in the hydrothermal subsurface biosphere will become increasingly clear.

## Description of candidatus “Polytropus marinifundus”

*Candidatus* Polytropus (Po.ly.tro’pus. N.L. masc. n. *Polytropus* (from Gr. masc. adj. *polytropus*) one that turns many ways, versatile). *Candidatus* Polytropus marinifundi (ma.ri.ni.fun’di. L. adj. *marinus* of the sea, marine; L. masc. n. *fundus* the bottom; N.L. gen. n. *marinifundi* of/from the sea floor). “Polytropaceae” (Po.ly.tro’pa.ce.a.e N.L. n. “Polytropaceae” -entis, type genus of the family; suff. -aceae, ending to denote a family; N.L. masc. pl. n. Polytropaceae, the family of the genus “Polytropus”).

### Code availability

All unpublished software used in this publication are available on github; sequence_windower (https://github.com/geronimp/window_sequence); EnrichM (github.com/geronimp/enrichM); GenomeTreeTK (github.com/dparks1134/GenomeTreeTk); CompareM v0.0.22 (https://github.com/dparks1134/CompareM); fastacxxch.count.py, (github.com/geronimp/HandyScripts/blob/master/99_random/fastacxxch.count.py); FinishM (v0.0.7 github.com/wwood/finishm). GTDB-Tk (github.com/Ecogenomics/GTDBTk).

## Supplementary information

Supplementary Tables

Supplementary

Supplementary files doc

Supplementary_File_1

Supplementary_File_2

Supplementary_File_3

Supplementary_Fie_4

Supplementary_File_5

Supplementary_File_6

Supplementary_File_7

Supplementary_File_8

Supplementary_File_9

Supplementary_File_10

Supplementary_File_11

Supplementary_File_12

Supplementary_File_13

Supplementary_File_14

Supplementary_File_15

Supplementary_File_16

Supplementary_File_17

Supplementary_File_18

Supplementary_File_19

## Data Availability

The datasets analysed during the current study are available in the NCBI SRA, accession number SRR3723048. The Candidatus “Polytropus marinifundus” genome is available under the NCBI bioproject SAMN10474933. All alignments and phylogenetic trees are available in the Supplementary Files 1–20.
