# Supplementary material for: Divergent methyl-coenzyme M reductase genes in a deep-subseafloor Archaeoglobi
Source: ISME J. 2019 Jan 16;13(5):1269–79. doi: 10.1038/s41396-018-0343-2 (PMC6474303; doi:10.1038/s41396-018-0343-2)
Supplement: Supplementary file 1 — Supplementary Tables [file 41396_2018_343_MOESM1_ESM.docx]

## ***Supplementary Table 1.*** Genes encoding enzymes within *Ca.* P. marinifundus shown in **Figure 2.**

| **Gene** | **Fig 2****gene** | **Prokka annotation** | **Cluster** | **KO** | **PFam** | **TIGRfam** | **Common to****Bathym****Synt, Arch** | **Common to****Synt, Arch** | **Specific to****Ca. P. marinifundus****among Arch** |
| --- | --- | --- | --- | --- | --- | --- | --- | --- | --- |
| scaffold1_74 |  | Polysaccharide biosynthesis protein | cluster_65 | NA | PF01943.16 | TIGR02900 | FALSE | FALSE | TRUE |
| scaffold1_82 | fwdC | Molybdenum-containing formylmethanofuran dehydrogenase 1 subunit C | cluster_72 | K00202 | PF01493.18 | TIGR03122 | FALSE | FALSE | FALSE |
| scaffold1_83 | fwdA | dihydroorotase | cluster_73 | K00200 | PF07969.10 | TIGR03121 | FALSE | FALSE | FALSE |
| scaffold1_84 | fwdB | Formate dehydrogenase subunit alpha | cluster_1017 | K00201 | PF00384.21 | TIGR03129 | FALSE | FALSE | FALSE |
| scaffold1_85 | fwdD | Molybdenum-containing formylmethanofuran dehydrogenase 1 subunit C | cluster_74 | K00203 | PF01568.20 | TIGR01591 | FALSE | FALSE | FALSE |
| scaffold1_86 | porC | Pyruvate synthase subunit PorC | cluster_848 | K00172 | PF01558.17 | TIGR02175 | FALSE | FALSE | FALSE |
| scaffold1_87 | porD | Pyruvate synthase subunit PorD | cluster_75 | K00171 | PF00037.26 | TIGR02179 | FALSE | FALSE | FALSE |
| scaffold1_88 | porA | Pyruvate synthase subunit PorA | cluster_562 | K00169 | PF17147.3 | TIGR02176 | FALSE | FALSE | FALSE |
| scaffold1_89 | porB | Pyruvate synthase subunit PorB | cluster_76 | K00170 | PF02775.20 | TIGR02176 | FALSE | FALSE | FALSE |
| scaffold1_102 | sdhA | Fumarate reductase (CoM/CoB) subunit A | cluster_1247 | K00239 | PF00890.23 | TIGR01812 | FALSE | FALSE | FALSE |
| scaffold1_103 | sdhB | Fumarate reductase (CoM/CoB) subunit B | cluster_89 | K00240 | PF13085.5 | TIGR00384 | FALSE | FALSE | FALSE |
| scaffold1_104 | sdhC | Succinate dehydrogenase/Fumarate reductase transmembrane subunit | cluster_90 | K00241 | PF01127.21 | TIGR02970 | FALSE | FALSE | FALSE |
| scaffold1_105 | sdhD | Succinate dehydrogenase/Fumarate reductase transmembrane subunit | cluster_91 | K00242 | PF01127.21 | TIGR02970 | FALSE | FALSE | FALSE |
| scaffold1_111 |  | Transmembrane exosortase (Exosortase_EpsH) | cluster_828 | NA | PF09721.9 | TIGR04125 | FALSE | FALSE | TRUE |
| scaffold1_114 | MHC | Cytochrome c7 c | cluster_98 | NA | PF14522.5 | TIGR04257 | FALSE | FALSE | FALSE |
| scaffold1_128 |  | Transglutaminase-like superfamily protein | cluster_111 | NA | PF01841.18 | NA | FALSE | TRUE | TRUE |
| scaffold1_132 |  | dihydropyrimidine dehydrogenase subunit B | cluster_112 | K17828 | PF01180.20 | TIGR02700 | FALSE | FALSE | TRUE |
| scaffold1_148 |  | hypothetical protein | cluster_127 | NA | NA | NA | FALSE | FALSE | TRUE |
| scaffold1_164 |  | regulatory protein UhpC | cluster_139 | K08223 | PF07690.15 | TIGR00881 | FALSE | FALSE | TRUE |
| scaffold1_166 | paaH | 3-hydroxybutyryl-CoA dehydrogenase | cluster_707 | K00074 | PF02737.17 | TIGR02279 | FALSE | FALSE | FALSE |
| scaffold1_167 | crt | 3-hydroxypropionyl-coenzyme A dehydratase | cluster_1525 | K01715 | PF00378.19 | TIGR01929 | FALSE | FALSE | FALSE |
| scaffold1_173 | gck | Glycerate 2-kinase | cluster_146 | K11529 | PF13660.5 | NA | FALSE | FALSE | FALSE |
| scaffold1_180 | mtaA | Methylcobamide:CoM methyltransferase MtbA | cluster_151 | K14080 | PF01208.16 | TIGR01463 | FALSE | FALSE | FALSE |
| scaffold1_181 | mtmB | Monomethylamine methyltransferase MtmB1 | cluster_152 | K16176 | PF05369.11 | NA | FALSE | FALSE | FALSE |
| scaffold1_182 | mttC | Trimethylamine corrinoid protein | cluster_336 | K14084 | PF02310.18 | TIGR02370 | FALSE | FALSE | FALSE |
| scaffold1_186 | Acetate--CoA ligase [ADP-forming] | Acetate--CoA ligase [ADP-forming] II subunit alpha | cluster_910 | K09181 | PF13607.5 | TIGR02717 | FALSE | FALSE | FALSE |
| scaffold1_187 | Acetate--CoA ligase [ADP-forming] | Acetate--CoA ligase [ADP-forming] I subunit beta | cluster_909 | K09181 | PF13549.5 | TIGR01016 | FALSE | FALSE | FALSE |
| scaffold1_188 | MHC | Dissimilatory sulfite reductase | cluster_155 | NA | PF13435.5 | TIGR03508 | FALSE | FALSE | FALSE |
| scaffold1_217 | napH | quinol dehydrogenase membrane component | cluster_182 | K02574 | PF12801.6 | TIGR02163 | FALSE | FALSE | FALSE |
| scaffold1_223 |  | cob(I)alamin adenolsyltransferase/cobinamide ATP-dependent adenolsyltransferase | cluster_187 | K19221 | PF02572.14 | TIGR00708 | TRUE | TRUE | TRUE |
| scaffold1_224 | fumC | Fumarate hydratase class II | cluster_188 | K01679 | PF00206.19 | TIGR00839 | FALSE | TRUE | TRUE |
| scaffold1_249 | glk | D-allose kinase | cluster_208 | K00884 | PF00480.19 | TIGR00744 | FALSE | FALSE | FALSE |
| scaffold1_303 | mtd | F420-dependent methylenetetrahydromethanopterin dehydrogenase | cluster_257 | K00319 | PF01993.17 | NA | FALSE | FALSE | FALSE |
| scaffold1_311 |  | hypothetical protein | cluster_265 | NA | PF09884.8 | NA | FALSE | FALSE | TRUE |
| scaffold2_1 | bcd |  | cluster_1615 | K00248 | PF02770.18 | TIGR03207 | FALSE | FALSE | FALSE |
| scaffold2_2 | atoB | acetyl-CoA acetyltransferase | cluster_317 | K00626 | PF00108.22 | TIGR01930 | FALSE | FALSE | FALSE |
| scaffold2_40 | NOX-1 | Coenzyme A disulfide reductase | cluster_299 | K17870 | PF07992.13 | TIGR03385 | FALSE | FALSE | FALSE |
| scaffold2_41 |  | hypothetical protein | cluster_300 | K04085 | PF01206.16 | TIGR03527 | FALSE | FALSE | TRUE |
| scaffold2_47 | bcd | putative acyl-CoA dehydrogenase | cluster_180 | K00248 | PF00441.23 | TIGR03207 | FALSE | FALSE | FALSE |
| scaffold2_48 | mcmA2 | Dimethylamine corrinoid protein | cluster_1405 | K01849 | PF02310.18 | TIGR00640 | FALSE | FALSE | FALSE |
| scaffold2_49 | mcmA1 | methylmalonyl-CoA mutase | cluster_306 | K01848 | PF01642.21 | TIGR00641 | FALSE | FALSE | FALSE |
| scaffold2_59 | fumB | Putative L(+)-tartrate dehydratase subunit beta | cluster_312 | K01678 | PF05683.11 | TIGR00723 | FALSE | FALSE | FALSE |
| scaffold2_60 | fumA | fumarate hydratase | cluster_313 | K01677 | PF05681.13 | TIGR00722 | FALSE | FALSE | FALSE |
| scaffold2_67 | atoB | acetyl-CoA acetyltransferase | cluster_317 | K00626 | PF00108.22 | TIGR01930 | FALSE | FALSE | FALSE |
| scaffold2_81 |  | hypothetical protein | cluster_329 | NA | NA | NA | FALSE | TRUE | TRUE |
| scaffold2_83 |  | ParB-like nuclease domain protein | cluster_331 | NA | PF02195.17 | TIGR04285 | FALSE | FALSE | TRUE |
| scaffold2_87 | mtrH | Tetrahydromethanopterin S-methyltransferase subunit H | cluster_335 | K00584 | PF02007.17 | TIGR01114 | FALSE | FALSE | FALSE |
| scaffold2_91 | Corrinoid | Trimethylamine corrinoid protein | cluster_336 | K00548 | PF02310.18 | TIGR02370 | FALSE | FALSE | FALSE |
| scaffold2_99 | mer | 5%2C10-methylenetetrahydromethanopterin reductase | cluster_344 | K00320 | PF00296.19 | TIGR03555 | FALSE | FALSE | FALSE |
| scaffold2_100 |  | hypothetical protein | cluster_345 | NA | NA | NA | FALSE | TRUE | TRUE |
| scaffold2_104 | Fer | bidirectional hydrogenase complex protein HoxU | cluster_348 | K00123 | PF13510.5 | TIGR00194 | FALSE | FALSE | FALSE |
| scaffold2_105 | bcrA | BadF/BadG/BcrA/BcrD ATPase family protein | cluster_349 | K04114 | PF01869.19 | TIGR03192 | FALSE | FALSE | FALSE |
| scaffold2_106 | bcrD | BadF/BadG/BcrA/BcrD ATPase family protein | cluster_349 | K04115 | PF01869.19 | TIGR00241 | FALSE | FALSE | FALSE |
| scaffold2_107 | bcrB | 2-hydroxyglutaryl-CoA dehydratase%2C D-component | cluster_350 | K04113 | PF06050.12 | TIGR03191 | FALSE | FALSE | FALSE |
| scaffold2_108 | bcrC | 2-hydroxyglutaryl-CoA dehydratase%2C D-component | cluster_351 | K04112 | PF06050.12 | TIGR03190 | FALSE | FALSE | FALSE |
| scaffold2_137 | aa transporter | Periplasmic binding protein | cluster_135 | K01999 | PF13458.5 | TIGR03669 | FALSE | FALSE | FALSE |
| scaffold2_138 | aa transporter | branched-chain amino acid transporter permease subunit LivH | cluster_483 | K01997 | PF02653.15 | TIGR03409 | FALSE | FALSE | FALSE |
| scaffold2_139 | aa transporter | leucine/isoleucine/valine transporter permease subunit | cluster_482 | K01998 | PF02653.15 | TIGR03408 | FALSE | FALSE | FALSE |
| scaffold2_140 | aa transporter | putative branched-chain amino acid transport ATP-binding protein LivG | cluster_809 | K01995 | PF00005.26 | TIGR03411 | FALSE | FALSE | FALSE |
| scaffold2_141 | aa transporter | putative branched-chain amino acid transport ATP-binding protein LivG | cluster_809 | K01996 | PF00005.26 | TIGR03410 | FALSE | FALSE | FALSE |
| scaffold2_143 | mdh | Malate dehydrogenase | cluster_375 | K00024 | PF00056.22 | TIGR01771 | FALSE | FALSE | FALSE |
| scaffold2_150 |  | sulfur transfer complex subunit TusD | cluster_397 | K06039 | PF02635.14 | TIGR03010 | FALSE | FALSE | TRUE |
| scaffold2_158 | eno | Enolase | cluster_388 | K01689 | PF00113.21 | TIGR01060 | FALSE | FALSE | FALSE |
| scaffold2_166 |  | DNA-binding transcriptional repressor ArsR | cluster_396 | K03892 | PF01022.19 | NA | FALSE | FALSE | TRUE |
| scaffold2_169 | mtrH | Tetrahydromethanopterin S-methyltransferase subunit H | cluster_335 | K00584 | PF02007.17 | TIGR01114 | FALSE | FALSE | FALSE |
| scaffold2_224 |  | hypothetical protein | cluster_442 | NA | PF09885.8 | TIGR03271 | FALSE | TRUE | TRUE |
| scaffold2_225 |  | hypothetical protein | cluster_443 | NA | PF09875.8 | TIGR03272 | FALSE | FALSE | TRUE |
| scaffold2_226 | mcrC | Methyl-coenzyme M reductase operon protein C | cluster_444 | NA | PF04609.11 | TIGR03274 | FALSE | TRUE | TRUE |
| scaffold2_227 |  | hypothetical protein | cluster_445 | NA | PF09886.8 | TIGR03291 | FALSE | TRUE | TRUE |
| scaffold2_228 |  | hypothetical protein | cluster_446 | NA | NA | TIGR03268 | FALSE | TRUE | TRUE |
| scaffold2_229 |  | hypothetical protein | cluster_447 | NA | NA | NA | FALSE | FALSE | TRUE |
| scaffold2_230 | mcrA | Methyl-coenzyme M reductase I subunit alpha | cluster_1846 | K00399 | PF02249.16 | TIGR03256 | TRUE | TRUE | TRUE |
| scaffold2_231 | mcrG | Methyl-coenzyme M reductase II subunit gamma | cluster_1847 | K00402 | PF02240.15 | TIGR03259 | TRUE | TRUE | TRUE |
| scaffold2_232 | mcrB | Methyl-coenzyme M reductase II subunit beta | cluster_1845 | K00401 | PF02241.17 | TIGR03257 | TRUE | TRUE | TRUE |
| scaffold2_233 |  | hypothetical protein | cluster_1855 | NA | NA | NA | FALSE | FALSE | TRUE |
| scaffold2_234 |  | hypothetical protein | cluster_448 | NA | NA | NA | FALSE | FALSE | TRUE |
| scaffold2_235 |  | hypothetical protein | cluster_449 | NA | NA | NA | FALSE | FALSE | TRUE |
| scaffold2_239 | mcrC | Methyl-coenzyme M reductase I operon protein C | cluster_453 | K03421 | PF04609.11 | TIGR03264 | FALSE | FALSE | TRUE |
| scaffold3_1 |  | cytochrome c nitrite reductase pentaheme subunit | cluster_1298 | NA | PF14522.5 | TIGR03146 | FALSE | FALSE | TRUE |
| scaffold3_17 |  | Lipoate-protein ligase A subunit 1 | cluster_469 | K03800 | PF10437.8 | TIGR00545 | FALSE | FALSE | TRUE |
| scaffold3_18 |  | 4-oxalocrotonate tautomerase | cluster_470 | K01821 | PF01361.20 | TIGR00013 | FALSE | FALSE | TRUE |
| scaffold3_20 |  | putative sialic acid transporter | cluster_1641 | K08178 | PF07690.15 | TIGR00895 | FALSE | FALSE | TRUE |
| scaffold3_22 |  | Methylcobamide:CoM methyltransferase MtbA | cluster_151 | K01599 | PF01208.16 | TIGR01463 | FALSE | FALSE | TRUE |
| scaffold3_24 | mtrH | Tetrahydromethanopterin S-methyltransferase subunit H | cluster_335 | K00584 | PF02007.17 | TIGR01114 | FALSE | FALSE | FALSE |
| scaffold3_26 |  | dihydropteroate synthase | cluster_473 | K00796 | PF00809.21 | TIGR01496 | FALSE | FALSE | TRUE |
| scaffold3_32 | mer | 5%2C10-methylenetetrahydromethanopterin reductase | cluster_344 | K00320 | PF00296.19 | TIGR03555 | FALSE | FALSE | FALSE |
| scaffold3_44 | ftr | Formylmethanofuran--tetrahydromethanopterin formyltransferase | cluster_486 | K00672 | PF01913.17 | TIGR03119 | FALSE | FALSE | FALSE |
| scaffold3_54 | narG | Respiratory nitrate reductase subunit alpha | cluster_496 | K00370 | PF00384.21 | TIGR01580 | FALSE | FALSE | FALSE |
| scaffold3_55 | narH | Respiratory nitrate reductase subunit beta | cluster_497 | K00371 | PF13237.5 | TIGR01660 | FALSE | FALSE | FALSE |
| scaffold3_56 | narJ | Chaperone protein TorD | cluster_498 | K00373 | PF02613.14 | TIGR00684 | FALSE | FALSE | FALSE |
| scaffold3_57 | narI | Nitrate reductase gamma subunit | cluster_499 | K00374 | PF02665.13 | TIGR00351 | FALSE | FALSE | FALSE |
| scaffold3_58 | nitrate transporter | putative nitrate transporter | cluster_139 | K02575 | PF07690.15 | TIGR00886 | FALSE | FALSE | FALSE |
| scaffold3_82 |  | hypothetical protein | cluster_518 | NA | PF12838.6 | TIGR01582 | FALSE | FALSE | TRUE |
| scaffold3_83 |  | Tungsten-containing aldehyde ferredoxin oxidoreductase | cluster_519 | K03738 | PF01314.17 | NA | FALSE | FALSE | TRUE |
| scaffold3_102 |  | Chemotactic signal transduction system substrate-binding protein BasB | cluster_537 | K01999 | PF13458.5 | TIGR03407 | FALSE | FALSE | TRUE |
| scaffold4_1 | aa transporter | leucine/isoleucine/valine transporter permease subunit | cluster_482 | K01998 | PF02653.15 | TIGR03408 | FALSE | FALSE | FALSE |
| scaffold4_2 | aa transporter | branched-chain amino acid transporter permease subunit LivH | cluster_483 | K01997 | PF02653.15 | TIGR03409 | FALSE | FALSE | FALSE |
| scaffold4_3 | aa transporter | putative branched-chain amino acid transport ATP-binding protein LivG | cluster_809 | K01996 | PF00005.26 | TIGR03410 | FALSE | FALSE | FALSE |
| scaffold4_4 | aa transporter | putative branched-chain amino acid transport ATP-binding protein LivG | cluster_809 | K01995 | PF00005.26 | TIGR03411 | FALSE | FALSE | FALSE |
| scaffold4_5 | aa transporter | Chemotactic signal transduction system substrate-binding protein BasB | cluster_135 | K01999 | PF13458.5 | TIGR01409 | FALSE | FALSE | FALSE |
| scaffold4_10 | acs/or K04110 | Acetyl-coenzyme A synthetase | cluster_546 | K08295 | PF00501.27 | TIGR02262 | FALSE | FALSE | FALSE |
| scaffold4_11 | cutB | Glyceraldehyde dehydrogenase medium chain | cluster_547 | K11178 | PF03450.16 | TIGR03195 | FALSE | FALSE | FALSE |
| scaffold4_12 | cutA | Glyceraldehyde dehydrogenase large chain | cluster_548 | K04108 | PF01315.21 | TIGR03194 | FALSE | FALSE | FALSE |
| scaffold4_13 | cutC | Glyceraldehyde dehydrogenase small chain | cluster_549 | K03518 | PF01799.19 | TIGR03193 | FALSE | FALSE | FALSE |
| scaffold4_17 | tpiA | Triosephosphate isomerase | cluster_551 | K01803 | PF00121.17 | TIGR00419 | FALSE | FALSE | FALSE |
| scaffold4_47 | MCEE | Methylmalonyl-CoA epimerase | cluster_1286 | K05606 | PF13669.5 | TIGR03081 | FALSE | FALSE | FALSE |
| scaffold4_54 |  | Tungsten-containing aldehyde ferredoxin oxidoreductase | cluster_763 | K03738 | PF01314.17 | NA | FALSE | FALSE | TRUE |
| scaffold4_72 |  | Adenylate kinase | cluster_586 | K00939 | PF13207.5 | NA | FALSE | TRUE | TRUE |
| scaffold4_110 |  | hypothetical protein | cluster_622 | K03655 | PF01978.18 | TIGR01884 | FALSE | FALSE | TRUE |
| scaffold5_9 | hyaD | hydrogenase 2 maturation endopeptidase | cluster_631 | K03605 | PF01750.17 | TIGR00072 | FALSE | FALSE | FALSE |
| scaffold5_10 | hyaB | F420-non-reducing hydrogenase vhc subunit A | cluster_632 | K06281 | PF00374.18 | TIGR03295 | FALSE | FALSE | FALSE |
| scaffold5_11 | hyaA | F420-non-reducing hydrogenase subunit G | cluster_633 | K06282 | PF14720.5 | TIGR00391 | FALSE | FALSE | FALSE |
| scaffold5_12 | hyoE | thiamine monophosphate kinase | cluster_856 | K04655 | PF00586.23 | TIGR02124 | FALSE | FALSE | FALSE |
| scaffold5_13 | hypF | Acylphosphatase | cluster_634 | K04656 | PF00708.17 | TIGR00143 | FALSE | FALSE | FALSE |
| scaffold5_18 | fadD | 4-hydroxybutyrate--CoA ligase 2 | cluster_546 | K01897 | PF00501.27 | TIGR03205 | FALSE | FALSE | FALSE |
| scaffold5_20 |  | 2-isopropylmalate synthase | cluster_640 | K10977 | PF00682.18 | TIGR02090 | FALSE | FALSE | TRUE |
| scaffold5_25 | apt | Hypoxanthine/guanine phosphoribosyltransferase | cluster_644 | K00759 | PF00156.26 | TIGR01090 | FALSE | FALSE | FALSE |
| scaffold5_45 |  | Pyridoxamine 5'-phosphate oxidase | cluster_662 | NA | PF01243.19 | TIGR03618 | FALSE | FALSE | TRUE |
| scaffold5_65 |  | hypothetical protein | cluster_681 | NA | NA | TIGR04209 | FALSE | FALSE | TRUE |
| scaffold6_5 | mtrH | Tetrahydromethanopterin S-methyltransferase subunit H | cluster_335 | K00584 | PF02007.17 | TIGR01114 | FALSE | FALSE | FALSE |
| scaffold6_9 | mer | 5%2C10-methylenetetrahydromethanopterin reductase | cluster_344 | K00320 | PF00296.19 | TIGR03555 | FALSE | FALSE | TRUE |
| scaffold6_14 | mtrH | Tetrahydromethanopterin S-methyltransferase subunit H | cluster_335 | K00584 | PF02007.17 | TIGR01114 | FALSE | FALSE | FALSE |
| scaffold6_18 | hdrA | CoB--CoM heterodisulfide reductase iron-sulfur subunit A | cluster_690 | K03388 | PF07992.13 | TIGR01944 | FALSE | FALSE | FALSE |
| scaffold6_19 | fwdF | Polyferredoxin protein MvhB | cluster_691 | K00205 | PF00037.26 | TIGR01971 | FALSE | FALSE | FALSE |
| scaffold6_23 |  | hypothetical protein | cluster_695 | NA | NA | NA | FALSE | FALSE | TRUE |
| scaffold6_24 |  | hypothetical protein | cluster_696 | NA | NA | NA | FALSE | TRUE | TRUE |
| scaffold6_37 | paaH | 3-hydroxybutyryl-CoA dehydrogenase | cluster_707 | K00074 | PF02737.17 | TIGR02279 | FALSE | FALSE | FALSE |
| scaffold6_38 | atoB | acetyl-CoA acetyltransferase | cluster_317 | K00626 | PF00108.22 | TIGR01930 | FALSE | FALSE | FALSE |
| scaffold6_64 |  | Sugar-specific transcriptional regulator TrmB | cluster_731 | NA | PF01978.18 | TIGR01884 | FALSE | TRUE | TRUE |
| scaffold6_74 |  | protease 4 | cluster_740 | K04773 | PF01343.17 | TIGR00706 | FALSE | TRUE | TRUE |
| scaffold6_81 |  | acyl-CoA esterase | cluster_744 | K01055 | PF00561.19 | TIGR03695 | FALSE | FALSE | TRUE |
| scaffold6_87 |  | D-glyceraldehyde dehydrogenase (NADP(+)) | cluster_554 | K00135 | PF00171.21 | TIGR01804 | FALSE | FALSE | TRUE |
| scaffold6_92 |  | HTH-type sugar sensing transcriptional regulator TrmBL1 | cluster_752 | NA | PF01978.18 | TIGR01884 | FALSE | FALSE | TRUE |
| scaffold6_111 |  | [LysW]-aminoadipate semialdehyde/glutamate semialdehyde transaminase | cluster_1110 | K00823 | PF00202.20 | TIGR00707 | FALSE | FALSE | TRUE |
| scaffold6_114 |  | endonuclease III | cluster_765 | K07457 | PF00730.24 | TIGR01083 | FALSE | FALSE | TRUE |
| scaffold6_130 |  | Peptidase family M50 | cluster_781 | NA | PF02163.21 | TIGR03434 | FALSE | FALSE | TRUE |
| scaffold6_139 | porC | Pyruvate synthase subunit PorC | cluster_848 | K00172 | PF01558.17 | TIGR02175 | FALSE | FALSE | FALSE |
| scaffold6_140 | porD | Pyruvate synthase subunit PorD | cluster_563 | K00171 | PF00037.26 | TIGR02179 | FALSE | FALSE | FALSE |
| scaffold6_141 | porA | Pyruvate synthase subunit PorA | cluster_562 | K00169 | PF01855.18 | TIGR02176 | FALSE | FALSE | TRUE |
| scaffold6_142 | porB | Pyruvate synthase subunit PorB | cluster_76 | K00170 | PF02775.20 | TIGR02176 | FALSE | FALSE | FALSE |
| scaffold6_143 | atoB | acetyl-CoA acetyltransferase | cluster_317 | K00626 | PF00108.22 | TIGR01930 | FALSE | FALSE | FALSE |
| scaffold6_147 |  | ubiE/COQ5 methyltransferase family protein | cluster_787 | NA | PF01209.17 | TIGR02752 | FALSE | FALSE | TRUE |
| scaffold6_154 |  | hypothetical protein | cluster_791 | K07143 | PF06677.11 | NA | FALSE | FALSE | TRUE |
| scaffold6_167 | korD | Zinc-containing ferredoxin-1 | cluster_1206 | K00176 | PF00037.26 | TIGR02179 | FALSE | FALSE | FALSE |
| scaffold6_168 | korA | 2-oxoglutarate synthase subunit KorA | cluster_803 | K00174 | PF17147.3 | TIGR03710 | FALSE | FALSE | FALSE |
| scaffold6_169 | korB | 2-oxoglutarate synthase subunit KorB | cluster_804 | K00175 | PF02775.20 | TIGR02177 | FALSE | FALSE | FALSE |
| scaffold6_170 | korC | 2-oxoglutarate synthase subunit KorC | cluster_848 | K00177 | PF01558.17 | TIGR03710 | FALSE | FALSE | FALSE |
| scaffold6_171 | sucC | succinyl-CoA synthetase subunit beta | cluster_805 | K01903 | PF08442.9 | TIGR01016 | FALSE | FALSE | FALSE |
| scaffold6_172 | sucD | Succinate--CoA ligase [ADP-forming] subunit alpha | cluster_1488 | K01902 | PF02629.18 | TIGR01019 | FALSE | FALSE | FALSE |
| scaffold6_181 | gudB | Glutamate dehydrogenase | cluster_834 | K00260 | PF00208.20 | NA | FALSE | FALSE | FALSE |
| scaffold6_204 |  | hypothetical protein | cluster_833 | NA | NA | NA | FALSE | TRUE | TRUE |
| scaffold6_206 |  | iron-sulfur cluster assembly protein | cluster_835 | K13628 | PF01521.19 | TIGR00049 | FALSE | FALSE | TRUE |
| scaffold6_214 |  | Putative zinc- or iron-chelating domain protein | cluster_842 | NA | PF03692.14 | NA | FALSE | FALSE | TRUE |
| scaffold6_215 |  | Pyridoxal 5'-phosphate synthase subunit PdxT | cluster_843 | NA | PF09825.8 | TIGR03800 | FALSE | FALSE | TRUE |
| scaffold6_221 |  | Indolepyruvate oxidoreductase subunit IorA | cluster_710 | K00179 | PF00037.26 | TIGR03336 | FALSE | FALSE | TRUE |
| scaffold6_228 |  | Tungsten-containing aldehyde ferredoxin oxidoreductase | cluster_763 | K03738 | PF01314.17 | NA | FALSE | FALSE | TRUE |
| scaffold6_269 | hdrD | CoB--CoM heterodisulfide reductase iron-sulfur subunit D | cluster_1838 | K08264 | PF02754.15 | TIGR03288 | FALSE | FALSE | FALSE |
| scaffold6_273 |  | HTH-type transcriptional regulator LrpA | cluster_887 | K03718 | PF01037.20 | TIGR01884 | FALSE | FALSE | TRUE |
| scaffold6_288 |  | hypothetical protein | cluster_899 | NA | NA | TIGR01191 | FALSE | FALSE | TRUE |
| scaffold6_291 | CTH | hypothetical protein | cluster_902 | K01758 | PF01053.19 | TIGR01328 | FALSE | FALSE | FALSE |
| scaffold6_295 | pgk | Phosphoglycerate kinase | cluster_906 | K00927 | PF00162.18 | NA | FALSE | FALSE | FALSE |
| scaffold6_300 | K01905 | Acetate--CoA ligase [ADP-forming] II | cluster_909 | K01905 | PF13549.5 | TIGR02068 | FALSE | FALSE | FALSE |
| scaffold6_306 | pgi1 | glucose-6-phosphate isomerase | cluster_914 | K06859 | PF06560.10 | NA | FALSE | FALSE | FALSE |
| scaffold6_326 | hdrC | CoB--CoM heterodisulfide reductase iron-sulfur subunit C | cluster_933 | K03390 | PF13183.5 | TIGR03290 | FALSE | FALSE | FALSE |
| scaffold6_327 | hdrB | CoB--CoM heterodisulfide reductase subunit B | cluster_934 | K03389 | PF02754.15 | TIGR03288 | FALSE | FALSE | FALSE |
| scaffold7_21 |  | putative HTH-type transcriptional regulator | cluster_955 | K03718 | PF13404.5 | TIGR02937 | FALSE | FALSE | TRUE |
| scaffold7_42 |  | NADH pyrophosphatase | cluster_972 | K03574 | PF00293.27 | TIGR02705 | FALSE | TRUE | TRUE |
| scaffold7_58 |  | cytochrome c-type biogenesis protein CcmE | cluster_666 | K02197 | PF03100.14 | NA | FALSE | FALSE | TRUE |
| scaffold7_73 |  | hypothetical protein | cluster_1821 | NA | NA | NA | FALSE | FALSE | TRUE |
| scaffold8_15 | glnA | Glutamine synthetase | cluster_1234 | K01915 | PF00120.23 | TIGR00653 | FALSE | FALSE | FALSE |
| scaffold8_27 | korB | 2-oxoglutarate synthase subunit KorB | cluster_804 | K00175 | PF12367.7 | TIGR02177 | FALSE | FALSE | FALSE |
| scaffold8_28 | korA | 2-oxoglutarate synthase subunit KorA | cluster_1014 | K00174 | PF01558.17 | TIGR03710 | FALSE | FALSE | FALSE |
| scaffold8_33 | cdhA | Acetyl-CoA decarbonylase/synthase complex subunit alpha 2 | cluster_1019 | K00192 | PF03063.19 | TIGR00314 | FALSE | FALSE | FALSE |
| scaffold8_34 | cdhB | Acetyl-CoA decarbonylase/synthase complex subunit epsilon 2 | cluster_1020 | K00195 | PF02552.15 | TIGR00315 | FALSE | FALSE | FALSE |
| scaffold8_35 | cdhC | Acetyl-CoA decarbonylase/synthase complex subunit beta | cluster_1021 | K00193 | PF03598.14 | TIGR00316 | FALSE | FALSE | FALSE |
| scaffold8_36 | cooC | protochlorophyllide reductase iron-sulfur ATP-binding protein | cluster_1022 | K07321 | PF01656.22 | TIGR01969 | FALSE | FALSE | FALSE |
| scaffold8_37 | cdhD | Acetyl-CoA decarbonylase/synthase complex subunit delta | cluster_1023 | K00194 | PF03599.15 | TIGR00381 | FALSE | FALSE | FALSE |
| scaffold8_38 | cdhE | Acetyl-CoA decarbonylase/synthase complex subunit gamma | cluster_1024 | K00197 | PF03599.15 | TIGR00381 | FALSE | FALSE | FALSE |
| scaffold8_47 | atpI | V-type ATP synthase subunit I | cluster_1032 | K02123 | PF01496.18 | NA | FALSE | FALSE | FALSE |
| scaffold8_48 | atpK | V-type ATP synthase subunit K | cluster_1033 | K02124 | PF00137.20 | TIGR01100 | FALSE | FALSE | FALSE |
| scaffold8_49 | atpE | V-type ATP synthase subunit E | cluster_1034 | K02121 | PF01991.17 | TIGR03321 | FALSE | FALSE | FALSE |
| scaffold8_50 | atpC | V-type ATP synthase subunit C | cluster_1035 | K02119 | PF01992.15 | TIGR02923 | FALSE | FALSE | FALSE |
| scaffold8_51 | atpF | V-type ATP synthase subunit F | cluster_1036 | K02122 | PF01990.16 | TIGR01101 | FALSE | FALSE | FALSE |
| scaffold8_52 | atpA | V-type ATP synthase alpha chain | cluster_1037 | K02117 | PF00006.24 | TIGR01043 | FALSE | FALSE | FALSE |
| scaffold8_53 | atpB | V-type ATP synthase beta chain | cluster_1037 | K02118 | PF00006.24 | TIGR01041 | FALSE | FALSE | FALSE |
| scaffold8_54 | atpD | V-type ATP synthase subunit D | cluster_1038 | K02120 | PF01813.16 | TIGR00309 | FALSE | FALSE | FALSE |
| scaffold8_82 |  | hypothetical protein | cluster_1065 | NA | PF04306.12 | TIGR03789 | FALSE | FALSE | TRUE |
| scaffold8_110 |  | hypothetical protein | cluster_1093 | NA | PF15919.4 | NA | FALSE | FALSE | TRUE |
| scaffold8_121 | mer | 5%2C10-methylenetetrahydromethanopterin reductase | cluster_344 | K00320 | PF00296.19 | TIGR03617 | FALSE | FALSE | FALSE |
| scaffold8_125 |  | hypothetical protein | cluster_1106 | NA | NA | NA | FALSE | TRUE | TRUE |
| scaffold8_130 | rbsk | putative sugar kinase | cluster_1111 | K00852 | PF00294.23 | TIGR04382 | FALSE | FALSE | FALSE |
| scaffold8_137 |  | Pyridoxamine 5'-phosphate oxidase | cluster_1118 | K07005 | PF12900.6 | TIGR03668 | FALSE | FALSE | TRUE |
| scaffold8_151 |  | hypothetical protein | cluster_1131 | NA | PF07758.10 | NA | FALSE | TRUE | TRUE |
| scaffold8_157 | asnB | asparagine synthetase B | cluster_1137 | K01953 | PF00733.20 | TIGR01536 | FALSE | FALSE | FALSE |
| scaffold8_171 |  | hypothetical protein | cluster_1147 | K04093 | PF01817.20 | TIGR01791 | FALSE | TRUE | TRUE |
| scaffold8_177 | maeA | bifunctional malic enzyme oxidoreductase/phosphotransacetylase | cluster_1152 | K00027 | PF00390.18 | NA | FALSE | FALSE | FALSE |
| scaffold8_187 |  | pyruvate oxidase | cluster_1638 | K00158 | PF02776.17 | TIGR00118 | FALSE | FALSE | TRUE |
| scaffold8_189 | pyk | Pyruvate kinase | cluster_1163 | K00873 | PF00224.20 | TIGR01064 | FALSE | FALSE | FALSE |
| scaffold8_202 | MHC | Cytochrome c7 c | cluster_1174 | NA | PF14522.5 | TIGR03508 | FALSE | FALSE | FALSE |
| scaffold8_206 | cyto_b | putative respiratory nitrate reductase subunit cytochrome b-561 | cluster_1177 | K03887 | PF00033.18 | TIGR02125 | FALSE | FALSE | FALSE |
| scaffold8_207 | cyto_b | putative respiratory nitrate reductase subunit cytochrome b-561 | cluster_1178 | K03888 | PF00032.16 | TIGR01156 | FALSE | FALSE | FALSE |
| scaffold8_208 | MHC | Dissimilatory sulfite reductase | cluster_1179 | NA | PF14522.5 | TIGR03508 | FALSE | FALSE | FALSE |
| scaffold8_211 | MHC, nrfH | Cytochrome c3 | cluster_1181 | K15876 | PF14537.5 | TIGR03153 | FALSE | FALSE | FALSE |
| scaffold8_212 | MHC, nrfH | Cytochrome c7 c | cluster_1181 | K15876 | PF14522.5 | TIGR03153 | FALSE | FALSE | FALSE |
| scaffold8_219 |  | hypothetical protein | cluster_1186 | NA | PF09873.8 | NA | FALSE | FALSE | TRUE |
| scaffold8_251 |  | putative Mg(2+) transport ATPase | cluster_1215 | K07507 | PF13194.5 | NA | FALSE | FALSE | TRUE |
| scaffold8_259 | rbi | Ribose 1%2C5-bisphosphate isomerase | cluster_1222 | K18237 | PF01008.16 | TIGR00511 | FALSE | FALSE | FALSE |
| scaffold8_261 | mch | Methenyltetrahydromethanopterin cyclohydrolase | cluster_1224 | K01499 | PF02289.15 | TIGR03120 | FALSE | FALSE | FALSE |
| scaffold9_3 | glnA | Glutamine synthetase | cluster_1234 | K01915 | PF00120.23 | TIGR00653 | FALSE | FALSE | FALSE |
| scaffold9_6 |  | glutamate synthase subunit alpha | cluster_1237 | K00265 | PF01645.16 | TIGR03966 | FALSE | FALSE | TRUE |
| scaffold9_9 | hdrD | CoB--CoM heterodisulfide reductase iron-sulfur subunit D | cluster_1838 | K08264 | PF02754.15 | TIGR03379 | FALSE | FALSE | FALSE |
| scaffold9_13 | fadD | Acetyl-coenzyme A synthetase | cluster_546 | K01897 | PF00501.27 | TIGR03205 | FALSE | FALSE | FALSE |
| scaffold9_18 |  | hypothetical protein | cluster_1244 | NA | PF01978.18 | NA | FALSE | TRUE | TRUE |
| scaffold9_48 |  | Thioredoxin | cluster_1749 | NA | PF13192.5 | TIGR00411 | FALSE | FALSE | TRUE |
| scaffold9_52 |  | 50S ribosomal protein L32 | cluster_1109 | NA | PF13248.5 | NA | FALSE | TRUE | TRUE |
| scaffold9_72 |  | phage shock protein PspA | cluster_1287 | K03969 | PF04012.11 | TIGR02977 | FALSE | FALSE | TRUE |
| scaffold9_73 |  | hypothetical protein | cluster_1288 | NA | NA | NA | FALSE | FALSE | TRUE |
| scaffold9_74 |  | hypothetical protein | cluster_1289 | NA | NA | NA | FALSE | FALSE | TRUE |
| scaffold9_78 |  | hypothetical protein | cluster_1293 | NA | NA | NA | TRUE | TRUE | TRUE |
| scaffold10_29 |  | Ribbon-helix-helix protein%2C copG family | cluster_1322 | K07746 | PF01402.20 | TIGR02606 | FALSE | FALSE | TRUE |
| scaffold10_35 |  | SNARE associated Golgi protein | cluster_1327 | NA | PF09335.10 | NA | FALSE | FALSE | TRUE |
| scaffold10_38 | atoB | acetyl-CoA acetyltransferase | cluster_317 | K00626 | PF00108.22 | TIGR01930 | FALSE | FALSE | FALSE |
| scaffold10_48 |  | Inosine-5'-monophosphate dehydrogenase | cluster_1336 | K04767 | PF00571.27 | TIGR01302 | FALSE | FALSE | TRUE |
| scaffold11_2 | prsA | Tripartite ATP-independent periplasmic transporter%2C DctM component | cluster_1349 | K00948 | PF06808.11 | TIGR02123 | FALSE | FALSE | FALSE |
| scaffold11_11 | argG | argininosuccinate synthase | cluster_1355 | K01940 | PF00764.18 | TIGR00032 | FALSE | FALSE | FALSE |
| scaffold11_13 |  | hypothetical protein | cluster_1356 | NA | NA | NA | FALSE | FALSE | TRUE |
| scaffold11_31 | NOX-1 | Coenzyme A disulfide reductase | cluster_1373 | K17870 | PF07992.13 | TIGR02374 | FALSE | FALSE | FALSE |
| scaffold11_40 |  | multidrug resistance protein D | cluster_139 | K08153 | PF07690.15 | TIGR00880 | FALSE | FALSE | TRUE |
| scaffold11_78 | atoB | acetyl-CoA acetyltransferase | cluster_317 | K00626 | PF02803.17 | TIGR01930 | FALSE | FALSE | FALSE |
| scaffold12_6 | mer | 5%2C10-methylenetetrahydromethanopterin reductase | cluster_344 | K00320 | PF00296.19 | TIGR03555 | FALSE | FALSE | FALSE |
| scaffold12_10 | hdrD | CoB--CoM heterodisulfide reductase iron-sulfur subunit D | cluster_1838 | K08264 | PF02754.15 | TIGR03379 | FALSE | FALSE | FALSE |
| scaffold12_12 | EtfA | hypothetical protein | cluster_1691 | K03521 | PF01012.20 | TIGR04503 | FALSE | FALSE | FALSE |
| scaffold12_13 | EtfB | electron transfer flavoprotein subunit YdiR | cluster_1690 | K03522 | PF00766.18 | NA | FALSE | FALSE | FALSE |
| scaffold12_14 | hdrD | CoB--CoM heterodisulfide reductase iron-sulfur subunit D | cluster_1614 | K08264 | PF02754.15 | TIGR03290 | FALSE | FALSE | TRUE |
| scaffold13_11 | gap2 | Glyceraldehyde-3-phosphate dehydrogenase | cluster_1425 | K00150 | PF01113.19 | TIGR01546 | FALSE | FALSE | FALSE |
| scaffold14_10 |  | hypothetical protein | cluster_1437 | NA | NA | TIGR04216 | FALSE | FALSE | TRUE |
| scaffold14_54 | ampp | AMP phosphorylase | cluster_1476 | K18931 | PF07831.12 | TIGR03327 | FALSE | FALSE | FALSE |
| scaffold14_68 | sucC | succinyl-CoA synthetase subunit beta | cluster_805 | K01903 | PF08442.9 | TIGR01016 | FALSE | FALSE | FALSE |
| scaffold14_69 | sucD | Succinate--CoA ligase [ADP-forming] subunit alpha | cluster_1488 | K01902 | PF02629.18 | TIGR01019 | FALSE | FALSE | FALSE |
| scaffold14_72 | hdrD | CoB--CoM heterodisulfide reductase iron-sulfur subunit D | cluster_1838 | K08264 | PF02754.15 | TIGR03379 | FALSE | FALSE | TRUE |
| scaffold14_76 |  | Sodium Bile acid symporter family protein | cluster_1492 | K03325 | PF01758.15 | TIGR00832 | FALSE | FALSE | TRUE |
| scaffold14_78 |  |  | cluster_511 | K04763 | PF00589.21 | TIGR02225 | FALSE | FALSE | TRUE |
| scaffold15_2 | fwdF | Polyferredoxin protein MvhB | cluster_1520 | K00205 | PF00037.26 | TIGR01971 | FALSE | FALSE | FALSE |
| scaffold15_3 | rbcL | Ribulose bisphosphate carboxylase | cluster_1521 | K01601 | PF00016.19 | TIGR03326 | FALSE | FALSE | FALSE |
| scaffold15_18 |  | hypothetical protein | cluster_1533 | NA | NA | NA | FALSE | FALSE | TRUE |
| scaffold15_20 | FBA | Fructose-1%2C6-bisphosphate aldolase/phosphatase | cluster_1535 | K01622 | PF01950.15 | NA | FALSE | FALSE | FALSE |
| scaffold15_47 |  | hypothetical protein | cluster_1554 | NA | PF13620.5 | NA | FALSE | FALSE | TRUE |
| scaffold15_52 |  | hypothetical protein | cluster_275 | K07332 | PF00437.19 | TIGR03819 | FALSE | FALSE | TRUE |
| scaffold15_54 |  | Isopropylmalate/citramalate isomerase small subunit | cluster_1169 | K01704 | PF00694.18 | TIGR02087 | FALSE | FALSE | TRUE |
| scaffold16_2 | MHC | Cytochrome c7 c | cluster_1579 | NA | PF14522.5 | TIGR03153 | FALSE | FALSE | FALSE |
| scaffold16_3 | MHC | Cytochrome c7 c | cluster_1579 | NA | PF14522.5 | TIGR03508 | FALSE | FALSE | FALSE |
| scaffold16_4 | MHC | Cytochrome c7 c | cluster_1580 | NA | PF01835.18 | TIGR03508 | FALSE | FALSE | FALSE |
| scaffold16_31 | hdrD | CoB--CoM heterodisulfide reductase iron-sulfur subunit D | cluster_1838 | K08264 | PF02754.15 | TIGR03379 | FALSE | FALSE | FALSE |
| scaffold16_32 | hdrE | CoB--CoM heterodisulfide reductase subunit E | cluster_1602 | K08265 | PF02665.13 | NA | FALSE | FALSE | TRUE |
| scaffold16_41 | argH | Fumarate hydratase class II | cluster_1611 | K01755 | PF14698.5 | TIGR00838 | FALSE | FALSE | FALSE |
| scaffold16_44 | Fe-S Oxidoreductase | CoB--CoM heterodisulfide reductase iron-sulfur subunit D | cluster_1614 | K08264 | PF02754.15 | TIGR03379 | FALSE | FALSE | FALSE |
| scaffold16_45 | acd |  | cluster_1615 | K00249 | PF02770.18 | TIGR03207 | FALSE | FALSE | FALSE |
| scaffold17_12 |  | hypothetical protein | cluster_1626 | NA | NA | NA | FALSE | FALSE | TRUE |
| scaffold18_14 | ala | Alanine dehydrogenase | cluster_923 | K19244 | PF02423.14 | TIGR02371 | FALSE | FALSE | FALSE |
| scaffold18_22 |  | drug efflux system protein MdtG | cluster_1641 | K08223 | PF07690.15 | TIGR00881 | FALSE | FALSE | TRUE |
| scaffold18_43 | hdrD | CoB--CoM heterodisulfide reductase iron-sulfur subunit D | cluster_1838 | K08264 | PF02754.15 | TIGR00273 | FALSE | FALSE | FALSE |
| scaffold18_44 |  | hypothetical protein | cluster_1652 | NA | PF04256.11 | NA | FALSE | FALSE | TRUE |
| scaffold18_62 |  | hypothetical protein | cluster_1669 | NA | PF16238.4 | NA | FALSE | FALSE | TRUE |
| scaffold18_66 |  | hypothetical protein | cluster_1672 | NA | PF09897.8 | NA | FALSE | FALSE | TRUE |
| scaffold19_1 |  | Trehalose/maltose import ATP-binding protein MalK | cluster_1681 | K00400 | PF00005.26 | TIGR03269 | FALSE | FALSE | TRUE |
| scaffold19_2 | mcrD | Methyl-coenzyme M reductase I operon protein D | cluster_1680 | K03422 | PF02505.13 | TIGR03260 | FALSE | TRUE | TRUE |
| scaffold19_3 |  | Trehalose/maltose import ATP-binding protein MalK | cluster_1681 | K00400 | PF00005.26 | TIGR02315 | FALSE | FALSE | TRUE |
| scaffold19_4 |  | Coenzyme PQQ synthesis protein D | cluster_1682 | NA | PF05402.11 | TIGR03859 | FALSE | FALSE | TRUE |
| scaffold19_7 | gpmA | Small-conductance mechanosensitive channel MscMJ | cluster_1685 | K01834 | PF00924.17 | NA | FALSE | FALSE | FALSE |
| scaffold19_10 |  | hypothetical protein | cluster_1687 | NA | PF09874.8 | NA | FALSE | FALSE | TRUE |
| scaffold19_14 | EtfA | putative electron transfer flavoprotein FixB | cluster_1690 | K03522 | PF01012.20 | NA | FALSE | FALSE | FALSE |
| scaffold19_15 | EtfB | hypothetical protein | cluster_1691 | K03521 | PF01012.20 | TIGR04503 | FALSE | FALSE | FALSE |
| scaffold19_33 | NOX-1 | Coenzyme A disulfide reductase | cluster_299 | K17870 | PF07992.13 | TIGR03385 | FALSE | FALSE | FALSE |
| scaffold19_45 |  | hypothetical protein | cluster_1718 | NA | PF02635.14 | NA | FALSE | FALSE | TRUE |
| scaffold19_48 | hdrD | CoB--CoM heterodisulfide reductase iron-sulfur subunit D | cluster_1838 | K08264 | PF02754.15 | TIGR03379 | FALSE | FALSE | FALSE |
| scaffold20_2 |  | Energy-coupling factor transporter transmembrane protein EcfT | cluster_1728 | K02008 | PF02361.15 | TIGR02454 | FALSE | FALSE | TRUE |
| scaffold20_15 |  | hypothetical protein | cluster_1740 | NA | NA | NA | FALSE | FALSE | TRUE |
| scaffold20_17 |  | Cytochrome C and Quinol oxidase polypeptide I | cluster_1742 | K00404 | PF00115.19 | TIGR00780 | FALSE | FALSE | TRUE |
| scaffold20_28 |  | hypothetical protein | cluster_1753 | K09736 | PF01877.16 | NA | FALSE | FALSE | TRUE |
| scaffold20_45 |  | Rubrerythrin | cluster_1768 | NA | PF02915.16 | TIGR00754 | FALSE | FALSE | TRUE |
| scaffold20_52 |  | glycine cleavage system protein H | cluster_1773 | K02437 | PF01597.18 | TIGR00527 | FALSE | FALSE | TRUE |
| scaffold20_82 | aa transporter | leucine/isoleucine/valine transporter permease subunit | cluster_482 | K01998 | PF02653.15 | TIGR03408 | FALSE | FALSE | FALSE |
| scaffold20_83 | aa transporter | branched-chain amino acid transporter permease subunit LivH | cluster_483 | K01997 | PF02653.15 | TIGR03409 | FALSE | FALSE | FALSE |
| scaffold20_84 | aa transporter | Periplasmic binding protein | cluster_1801 | K01999 | PF13458.5 | TIGR01409 | FALSE | FALSE | FALSE |
| scaffold20_85 |  | Thermophilic metalloprotease (M29) | cluster_1802 | K19689 | PF02073.14 | NA | FALSE | FALSE | TRUE |
| scaffold20_95 | hdrD | CoB--CoM heterodisulfide reductase iron-sulfur subunit D | cluster_1838 | K08264 | PF02754.15 | TIGR03288 | FALSE | FALSE | TRUE |
| scaffold20_102 |  | AraC-like ligand binding domain protein | cluster_1812 | NA | PF02311.18 | NA | FALSE | FALSE | TRUE |
| scaffold20_105 | napD | NapD protein | cluster_1815 | NA | PF03927.12 | NA | FALSE | FALSE | FALSE |
| scaffold20_106 | napA | Formate dehydrogenase subunit alpha | cluster_353 | K02567 | PF00384.21 | TIGR01706 | FALSE | FALSE | FALSE |
| scaffold20_107 | MHC transmembrane? | Seven times multi-haem cytochrome CxxCH | cluster_155 | K10535 | PF13447.5 | TIGR03508 | FALSE | FALSE | FALSE |
| scaffold20_117 | fpoJ (nuoJ) | hypothetical protein | cluster_1824 | K00339 | PF00499.19 | NA | FALSE | FALSE | FALSE |
| scaffold20_118 | fpoK (nuoK) | NADH:ubiquinone oxidoreductase subunit K | cluster_1825 | K00340 | PF00420.23 | NA | FALSE | FALSE | FALSE |
| scaffold20_119 | fpoM (nuoM) | F(420)H(2) dehydrogenase subunit M | cluster_1826 | K00342 | PF00361.19 | TIGR01972 | FALSE | FALSE | FALSE |
| scaffold20_120 | fpoL (nuoL) | F(420)H(2) dehydrogenase subunit L | cluster_1827 | K00341 | PF00361.19 | TIGR01974 | FALSE | FALSE | FALSE |
| scaffold20_121 | fpoN (nuoN) | F(420)H(2) dehydrogenase subunit N | cluster_1828 | K00343 | PF00361.19 | TIGR01770 | FALSE | FALSE | FALSE |
| scaffold20_122 | fpoA (nuoA) | NADH dehydrogenase subunit A | cluster_1829 | K00330 | PF00507.18 | NA | FALSE | FALSE | FALSE |
| scaffold20_123 | fpoBC (nuoB) | F(420)H(2) dehydrogenase subunit B | cluster_1830 | K00331 | PF00329.18 | TIGR01957 | FALSE | FALSE | FALSE |
| scaffold20_124 | fpoD (nuoD) | F(420)H(2) dehydrogenase subunit D | cluster_1831 | K00333 | PF00346.18 | TIGR01962 | FALSE | FALSE | FALSE |
| scaffold20_125 | fpoH (nuoH) | F(420)H(2) dehydrogenase subunit H | cluster_1832 | K00337 | PF00146.20 | NA | FALSE | FALSE | FALSE |
| scaffold20_126 | fpoI (nuoI) | F(420)H(2) dehydrogenase subunit I | cluster_1833 | K00338 | PF00037.26 | TIGR01971 | FALSE | FALSE | FALSE |
| scaffold20_127 | fpoF | F(420)H(2) dehydrogenase subunit F | cluster_1834 | K00441 | PF12837.6 | TIGR03294 | FALSE | FALSE | FALSE |
| scaffold21_4 | hdrD | CoB--CoM heterodisulfide reductase iron-sulfur subunit D | cluster_1838 | K08264 | PF02754.15 | TIGR03379 | FALSE | FALSE | FALSE |
| scaffold21_6 |  | Dihydrolipoyl dehydrogenase | cluster_299 | K00382 | PF07992.13 | TIGR01350 | FALSE | FALSE | TRUE |
| scaffold21_8 |  | Small archaeal modifier protein 3 | cluster_849 | K03636 | PF02597.19 | TIGR01687 | FALSE | FALSE | TRUE |
| scaffold21_9 |  | hypothetical protein | cluster_1840 | NA | PF14196.5 | NA | FALSE | TRUE | TRUE |
| scaffold21_11 |  | hypothetical protein | cluster_1842 | NA | NA | NA | FALSE | FALSE | TRUE |
| scaffold21_13 |  | Nitrogenase component 1 type Oxidoreductase | cluster_1843 | K02586 | PF00148.18 | TIGR03282 | FALSE | TRUE | TRUE |
| scaffold21_14 |  | UDP-N-acetylmuramoylalanyl-D-glutamate--2%2C6-diaminopimelate ligase | cluster_1844 | NA | PF08245.11 | TIGR01085 | FALSE | TRUE | TRUE |
| scaffold21_16 | mcrB | Methyl-coenzyme M reductase II subunit beta | cluster_1845 | K00401 | PF02241.17 | TIGR03257 | FALSE | TRUE | TRUE |
| scaffold21_17 | mcrA | Methyl-coenzyme M reductase I subunit alpha | cluster_1846 | K00399 | PF02249.16 | TIGR03256 | FALSE | TRUE | TRUE |
| scaffold21_18 | mcrG | Methyl-coenzyme M reductase II subunit gamma | cluster_1847 | K00402 | PF02240.15 | TIGR03259 | TRUE | TRUE | TRUE |
| scaffold21_20 |  | Epoxyqueuosine reductase | cluster_1849 | K18979 | PF12838.6 | TIGR02486 | FALSE | FALSE | TRUE |
| scaffold21_21 |  | hypothetical protein | cluster_1850 | NA | PF01155.18 | NA | FALSE | FALSE | TRUE |
| scaffold21_23 |  | Polyferredoxin protein MvhB | cluster_1852 | K00196 | PF13187.5 | TIGR04105 | FALSE | FALSE | TRUE |
| scaffold21_25 |  | hypothetical protein | cluster_1854 | NA | PF09872.8 | TIGR03275 | TRUE | TRUE | TRUE |
| scaffold21_26 |  | hypothetical protein | cluster_1855 | NA | NA | TIGR01344 | FALSE | FALSE | TRUE |
| scaffold21_28 |  | hypothetical protein | cluster_1856 | NA | PF04945.12 | NA | FALSE | FALSE | TRUE |

## 
