# Supplementary material for: Divergent methyl-coenzyme M reductase genes in a deep-subseafloor Archaeoglobi
Source: ISME J. 2019 Jan 16;13(5):1269–79. doi: 10.1038/s41396-018-0343-2 (PMC6474303; doi:10.1038/s41396-018-0343-2)
Supplement: Supplementary file 2 — Supplementary [file 41396_2018_343_MOESM2_ESM.docx]

# **Supplementary** Figures


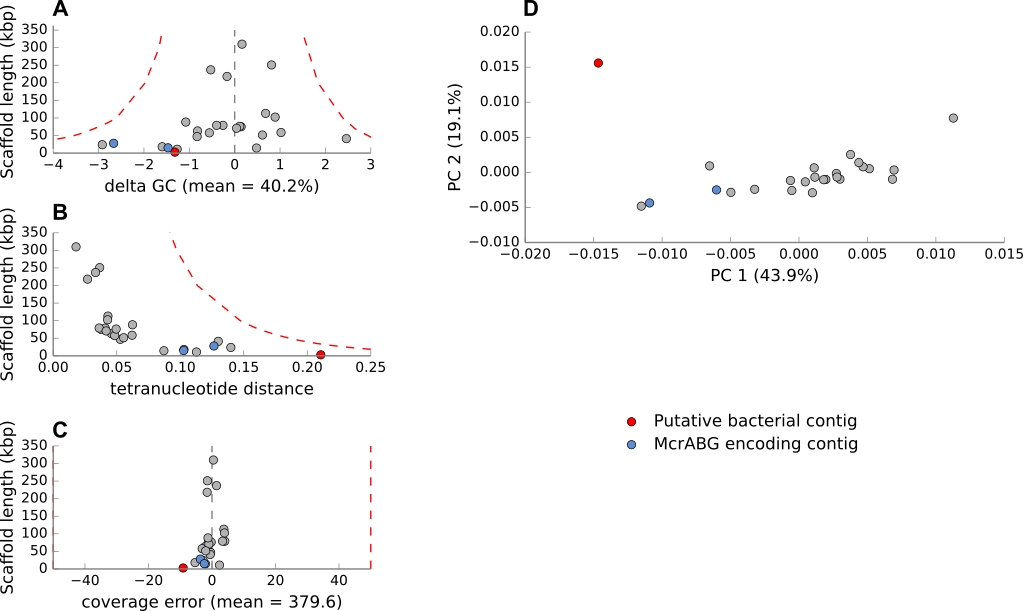


**Supplementary Figure 1.** RefineM analysis of the *Ca.* P. marinifundus MAG. **A)** %GC content **B)** tetranucleotide distance **C)** coverage error and **D)** tetranucleotide frequencies. Putative bacterial contigs are coloured Red. Red dashed lines represent the 98th percentile of expected distributions of these genomic features, as determined over a set of 5,656 trusted reference genomes.


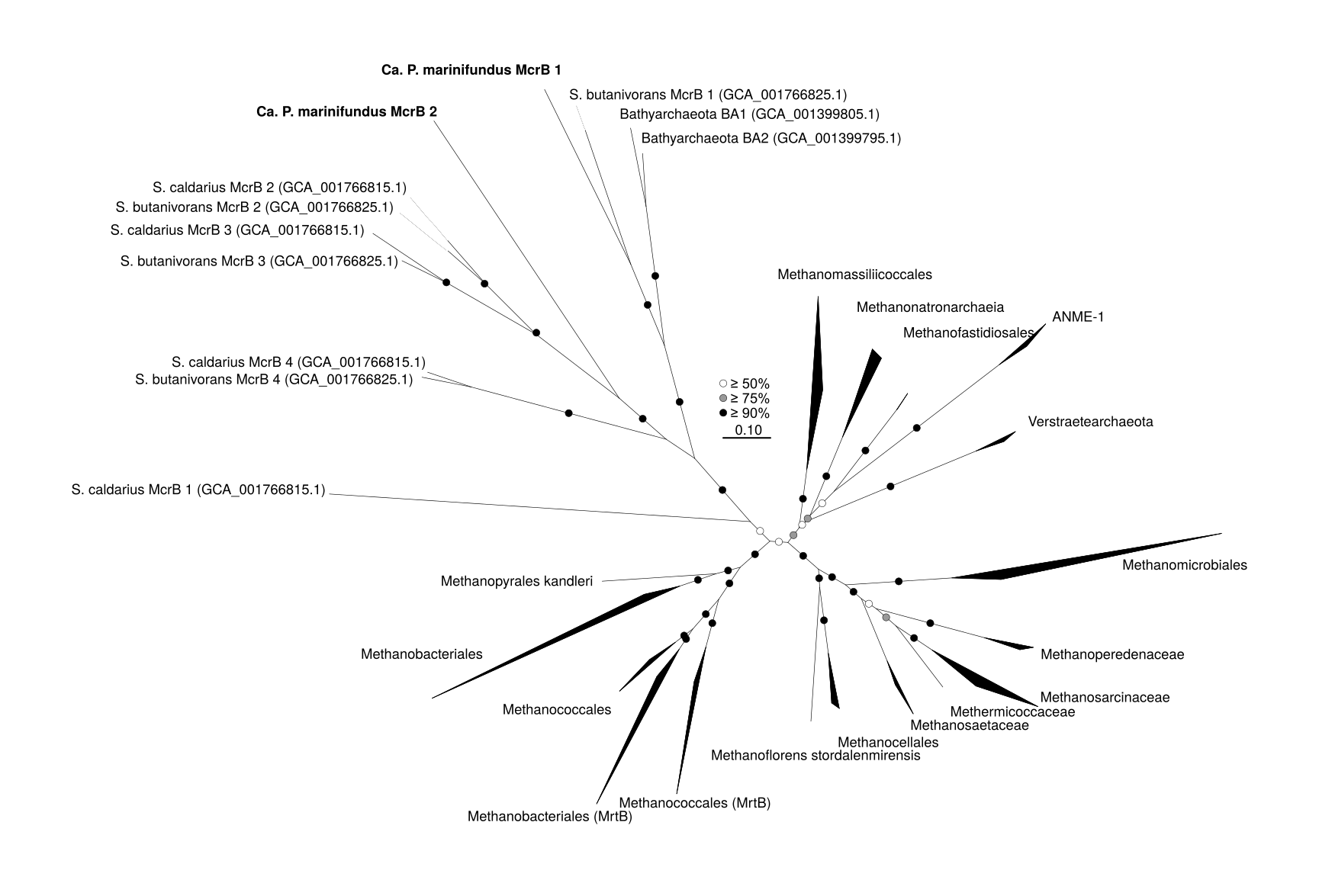


**Supplementary Figure 2.** FastTree constructed maximum likelihood tree of McrB sequences from high quality archaeal RefSeq genomes (release 80). Bootstrap support was generated from 100 replicates, and white, gray and black nodes represent ≥50%, ≥75% and ≥90% support, respectively.


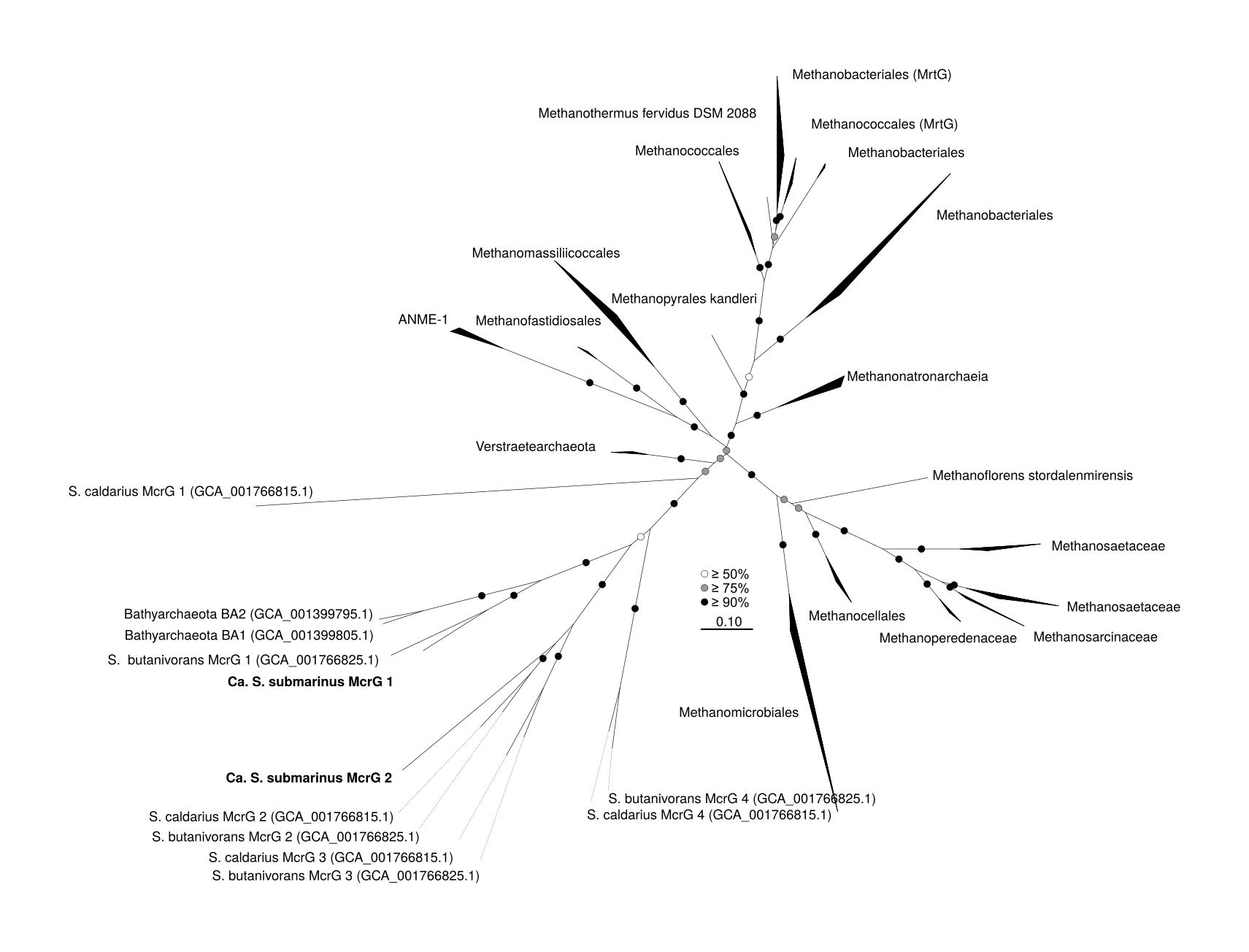


**Supplementary Figure 3.** FastTree constructed maximum likelihood tree of McrG sequences from high quality archaeal RefSeq genomes (release 80). Bootstrap support was generated from 100 replicates, and white, gray and black nodes represent ≥50%, ≥75% and ≥90% support, respectively.


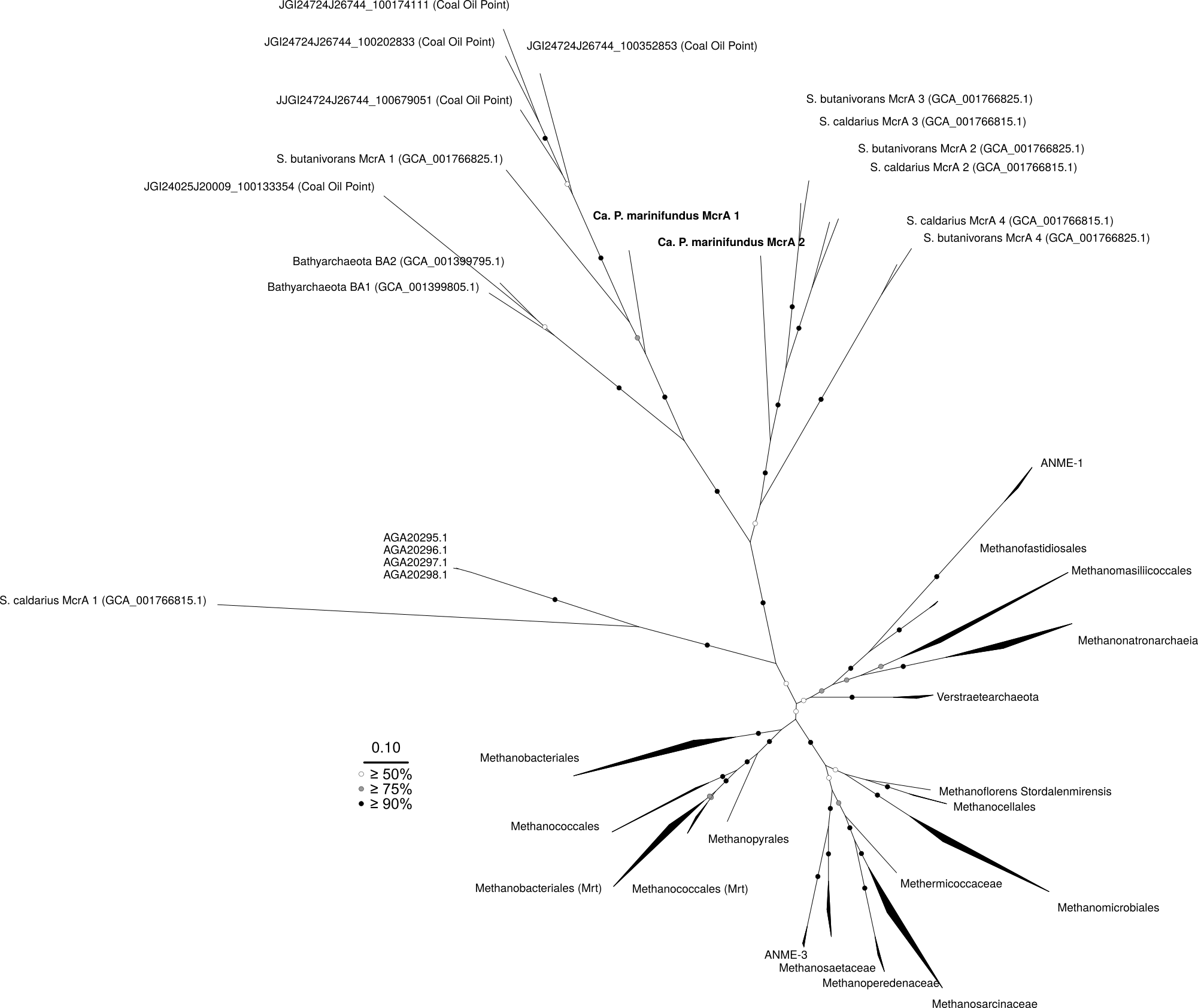


**Supplementary Figure 4.** As in Figure 1A, but constructed using IQ-TREE.

**
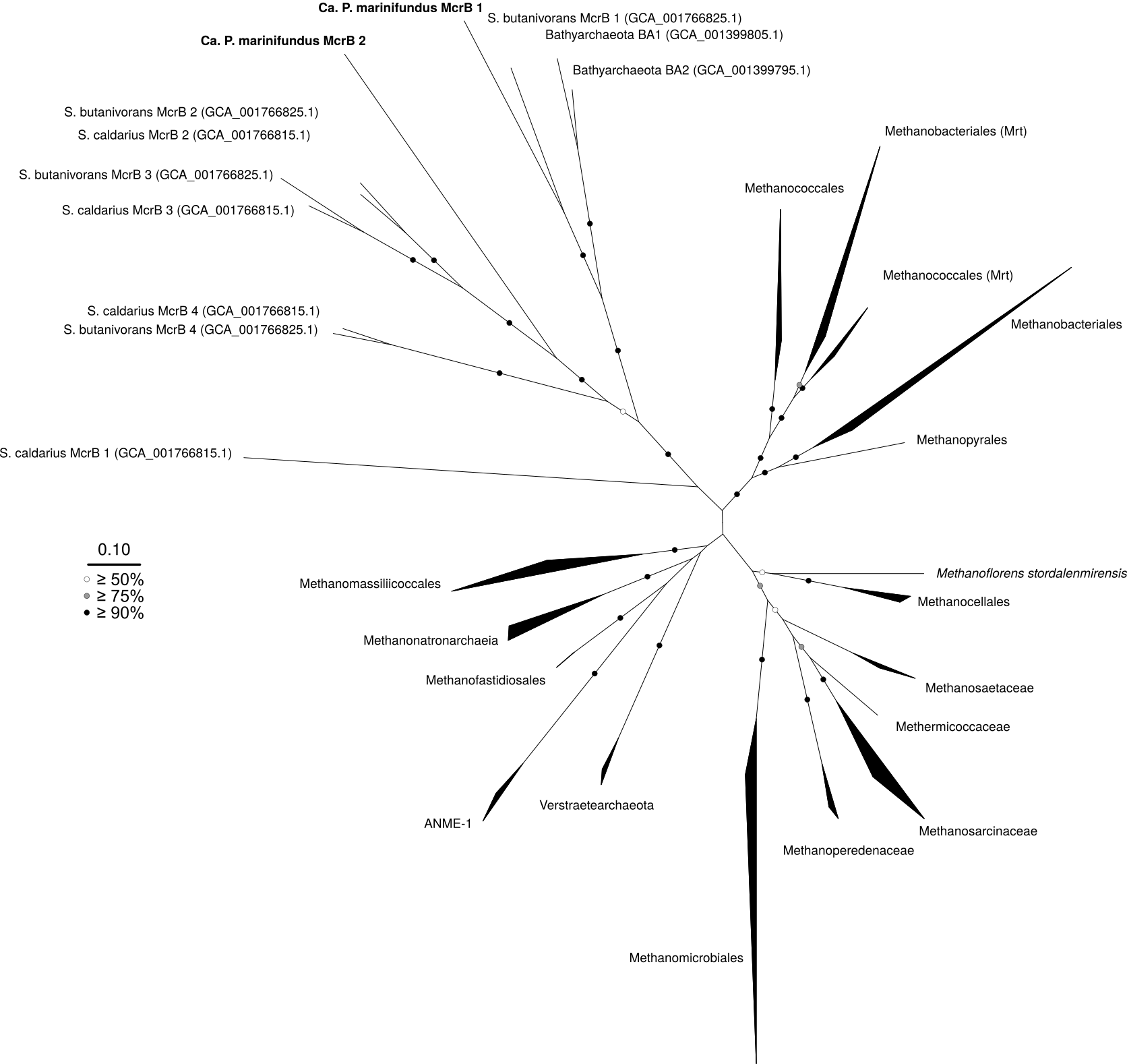
**

**Supplementary Figure 5.** As in Supplementary Figure 2, but constructed using IQ-TREE.

**
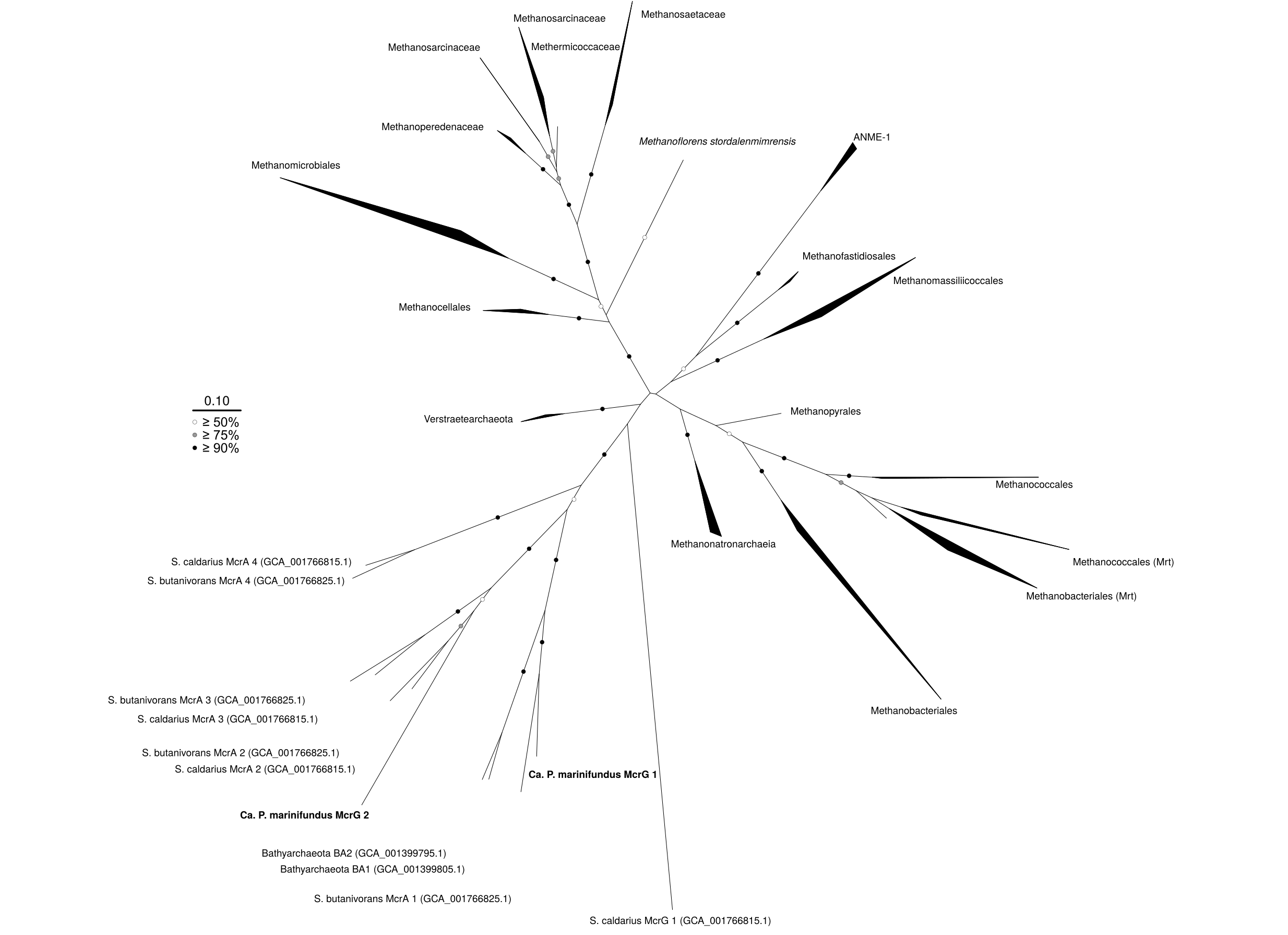
**

**Supplementary Figure 6.** As in Supplementary Figure 3, but constructed using IQ-TREE.

**
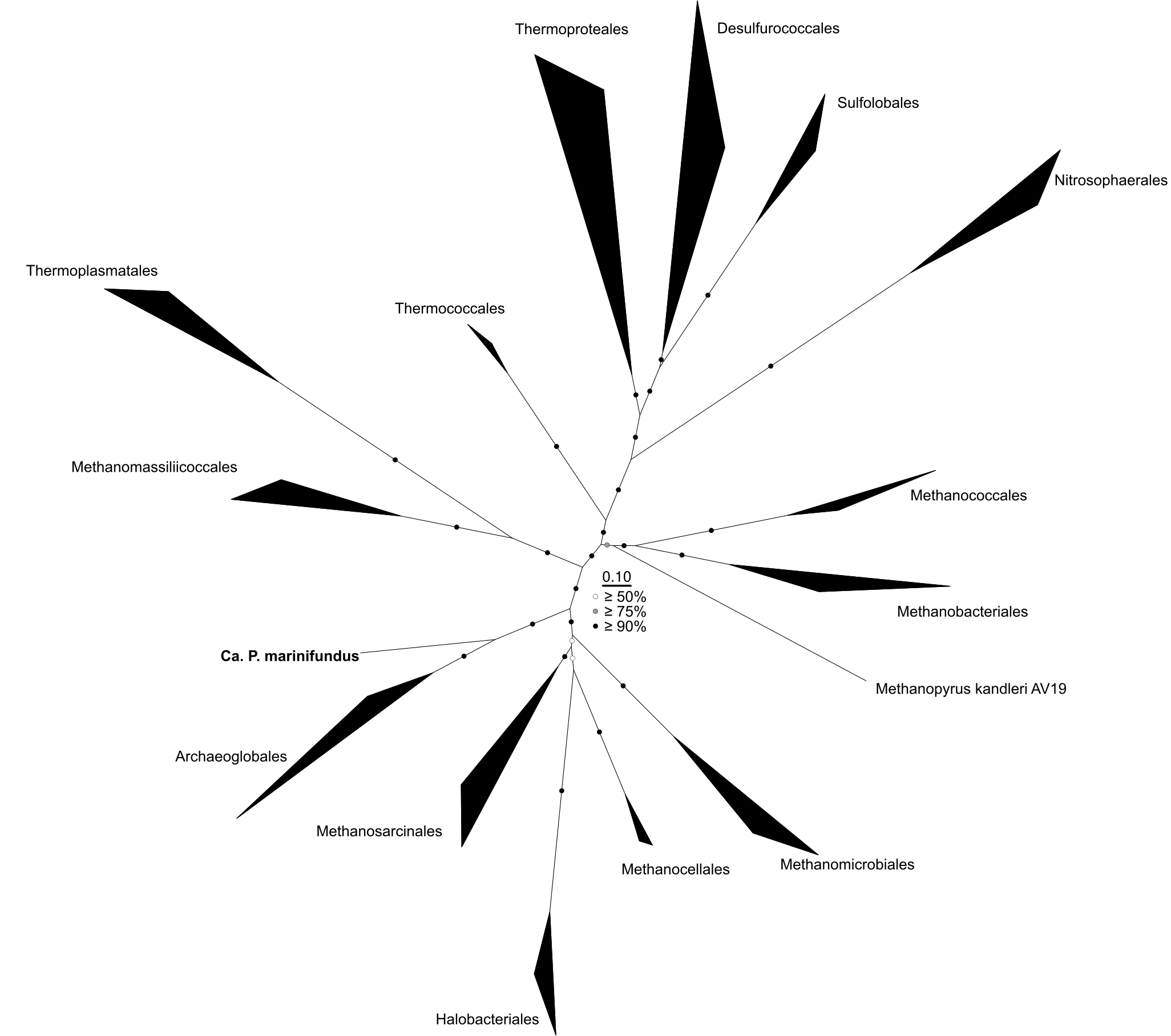
**

**Supplementary Figure 7.** As in Figure 1B, but constructed using IQ-TREE.


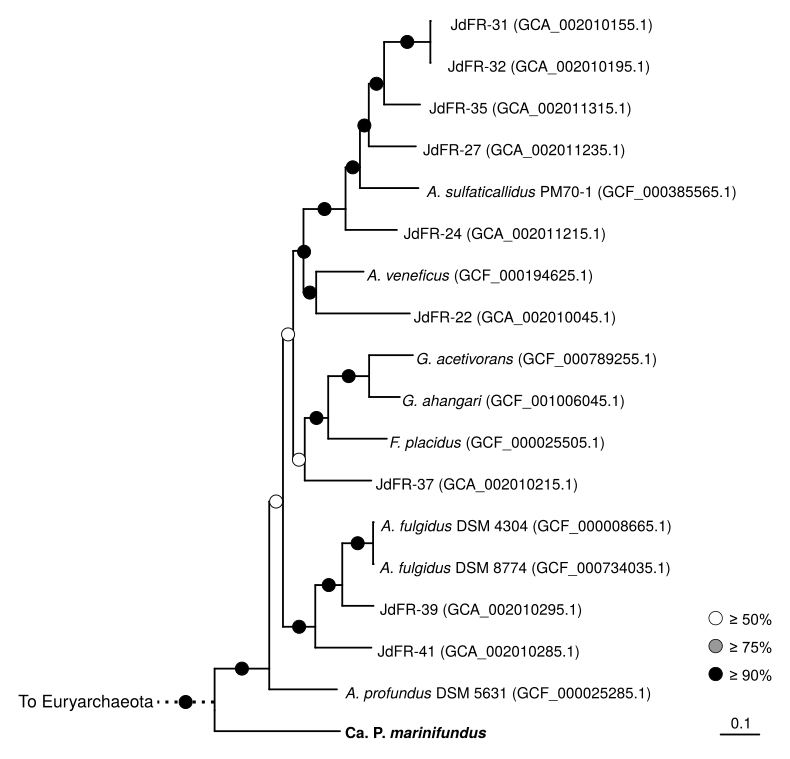


**Supplementary Figure 8.** FastTree constructed maximum likelihood genome tree of high quality archaeal RefSeq genomes (release 80) constructed using a concatenated alignment of 122 single copy archaeal marker genes. Bootstrap support was generated from 100 replicates, and white, gray and black nodes represent ≥50%, ≥75% and ≥90% support, respectively.


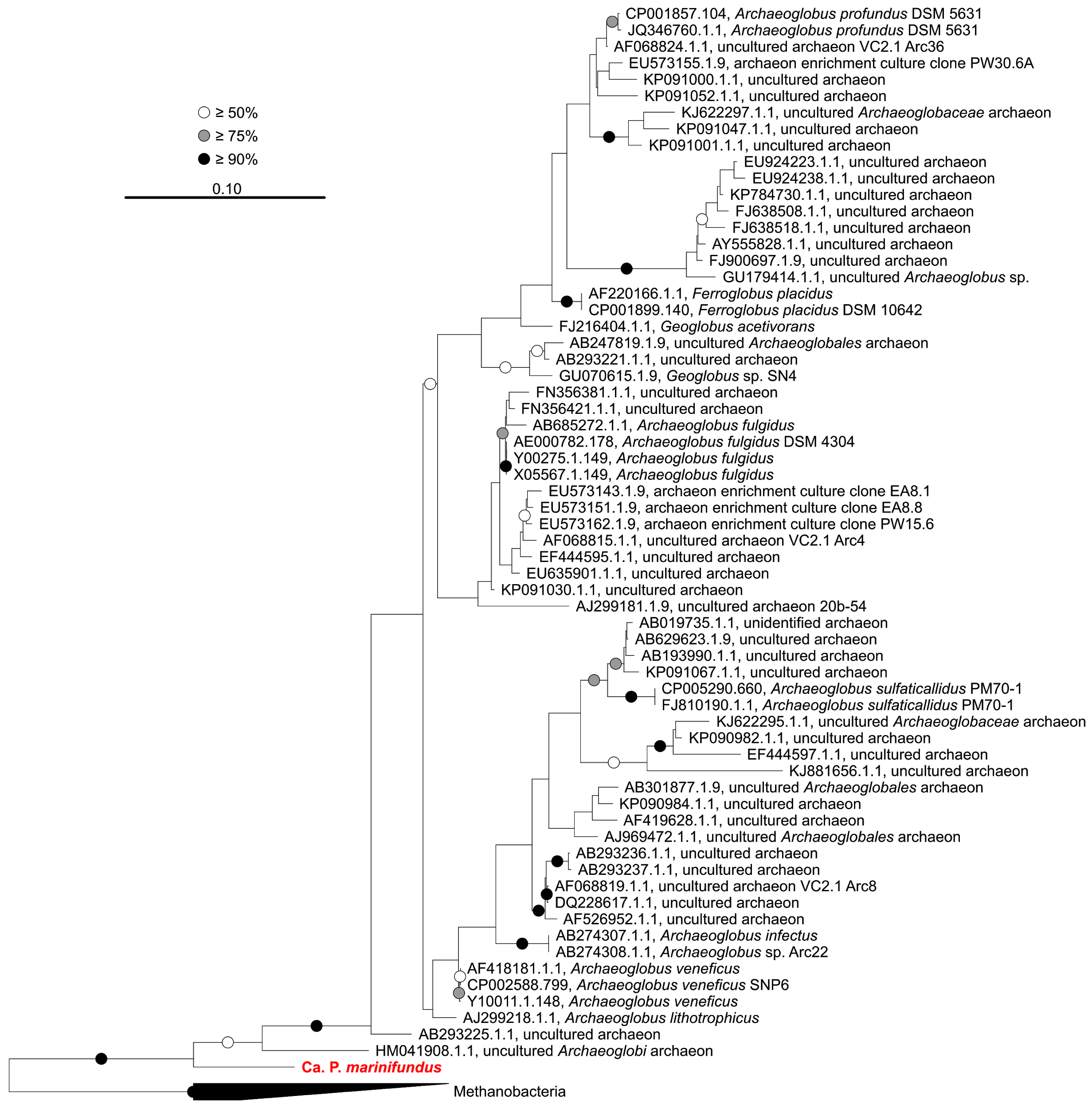


**Supplementary Figure 9.** FastTree constructed maximum likelihood tree of Archaeoglobi 16S rRNA sequences derived from the SILVA database (r132). Bootstrap support was generated from 100 replicates, and white, gray and black nodes represent ≥50%, ≥75% and ≥90% support, respectively.


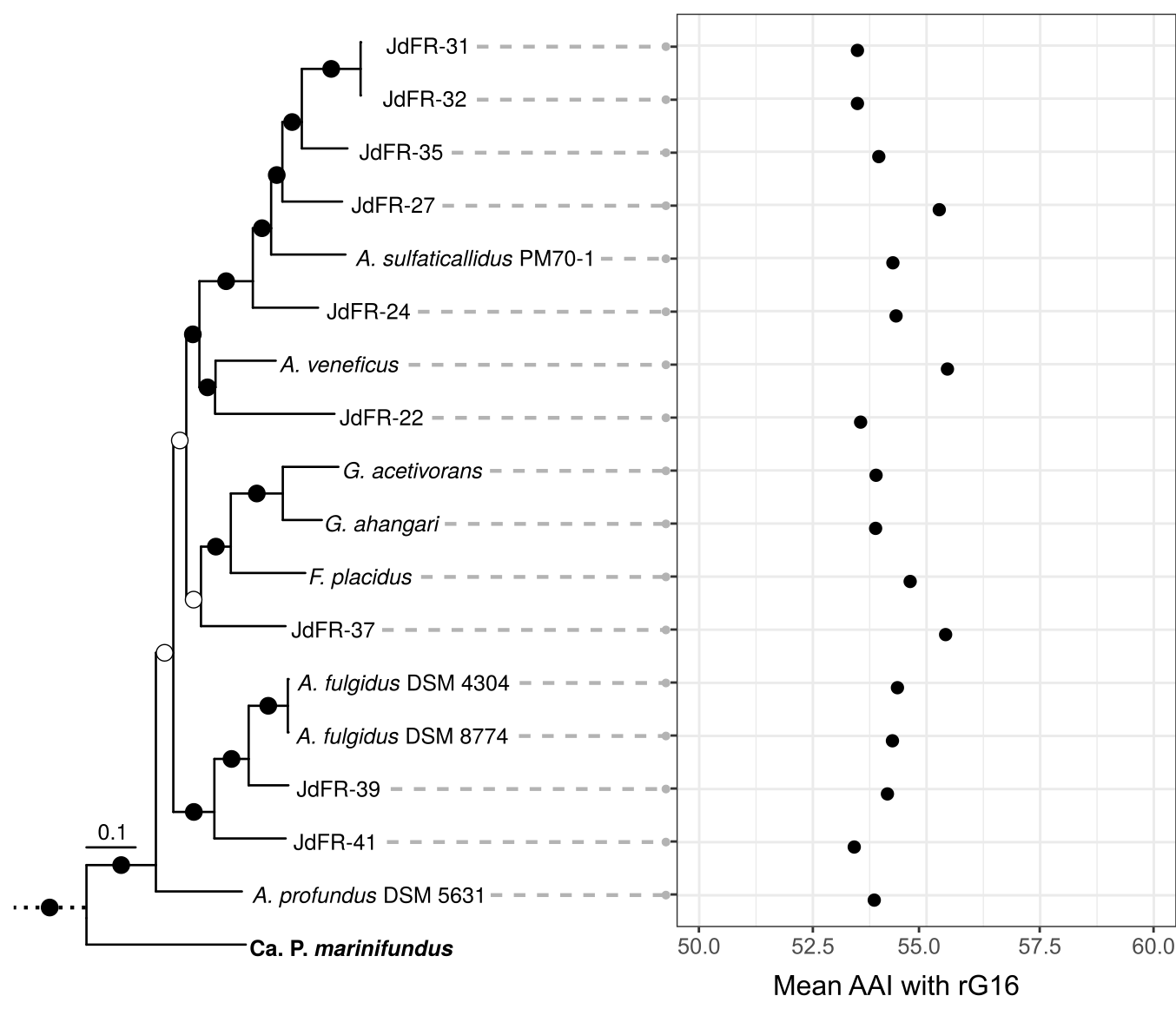


**Supplementary Figure 10.** Genome phylogeny as in Supplementary Figure 4. Amino acid identity (AAI) of orthologous genes was generated using the aai_wf of CompareM.

**
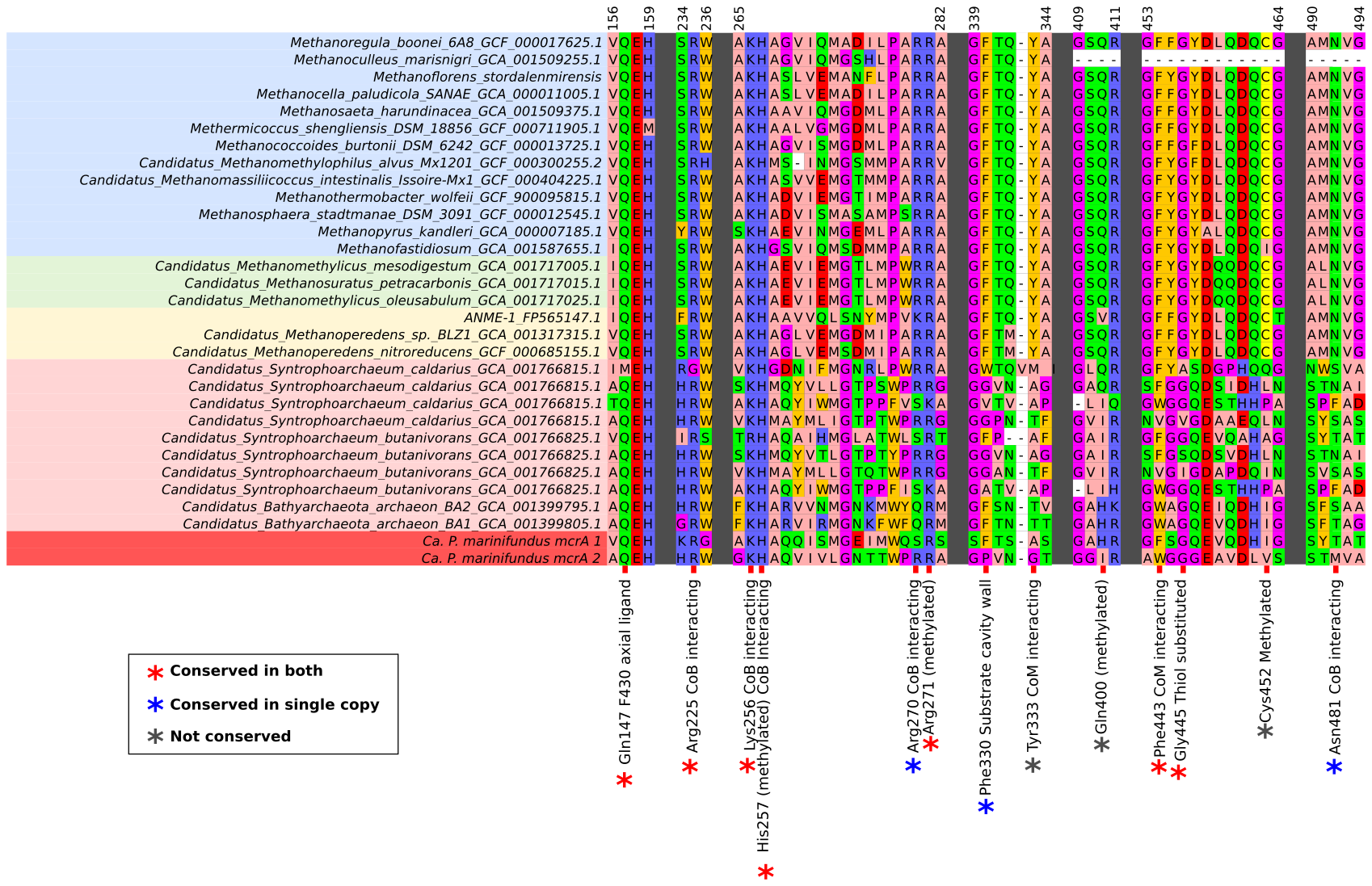
**

**Supplementary Figure 11.** Conservation in McrA sequences from high quality archaeal RefSeq genomes (release 80) of ligand cavity sites and F420, CoB, and CoM binding sites [[1]](https://paperpile.com/c/cShp06/gpOn4) in the *Ca.* P. marinifundus McrA proteins.


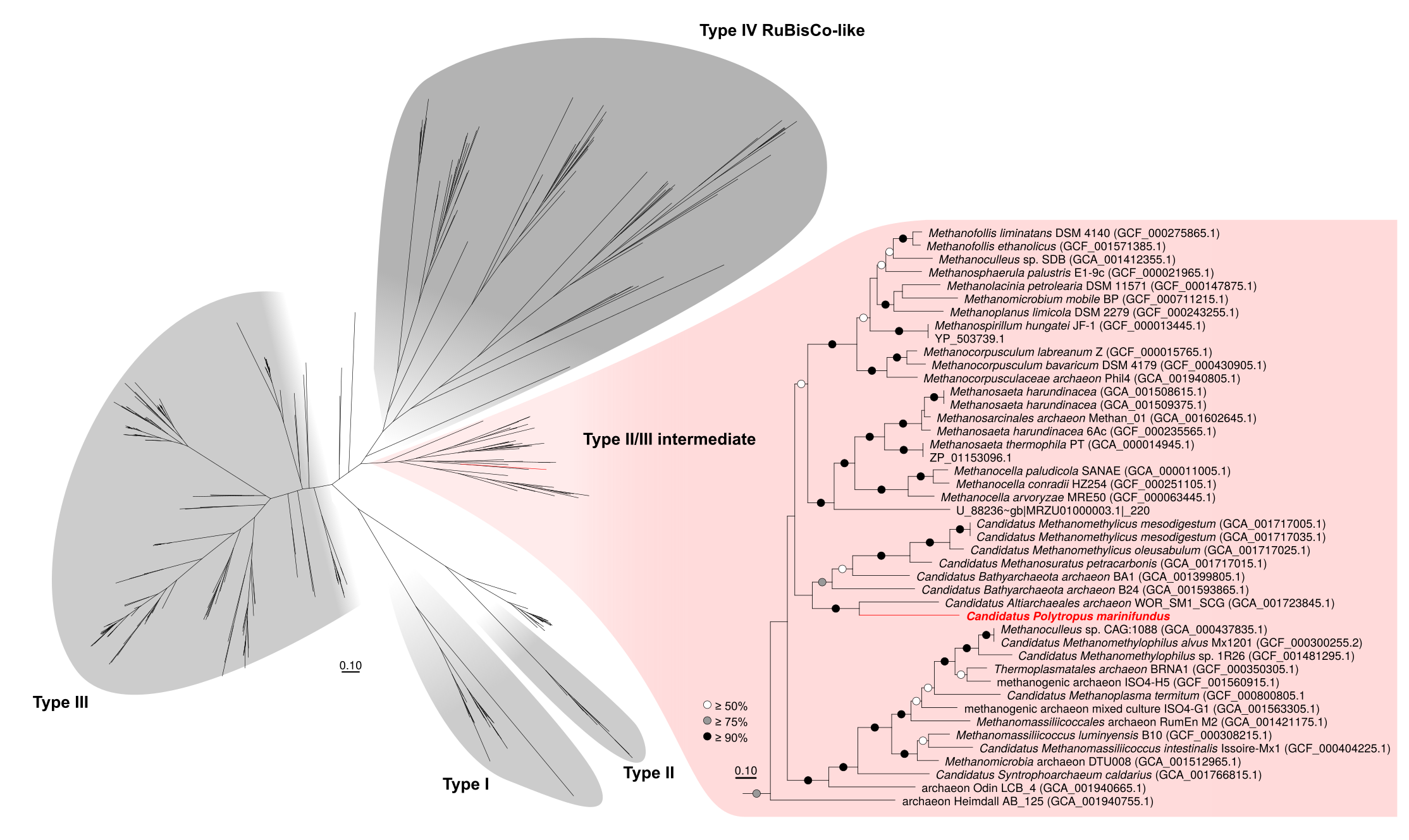


**Supplementary Figure 12.** FastTree constructed maximum likelihood tree of RuBisCo sequences extracted from high quality archaeal RefSeq genomes (release 80). Bootstrap support was generated from 100 replicates, and white, gray and black nodes represent ≥50%, ≥75% and ≥90% support, respectively.

**
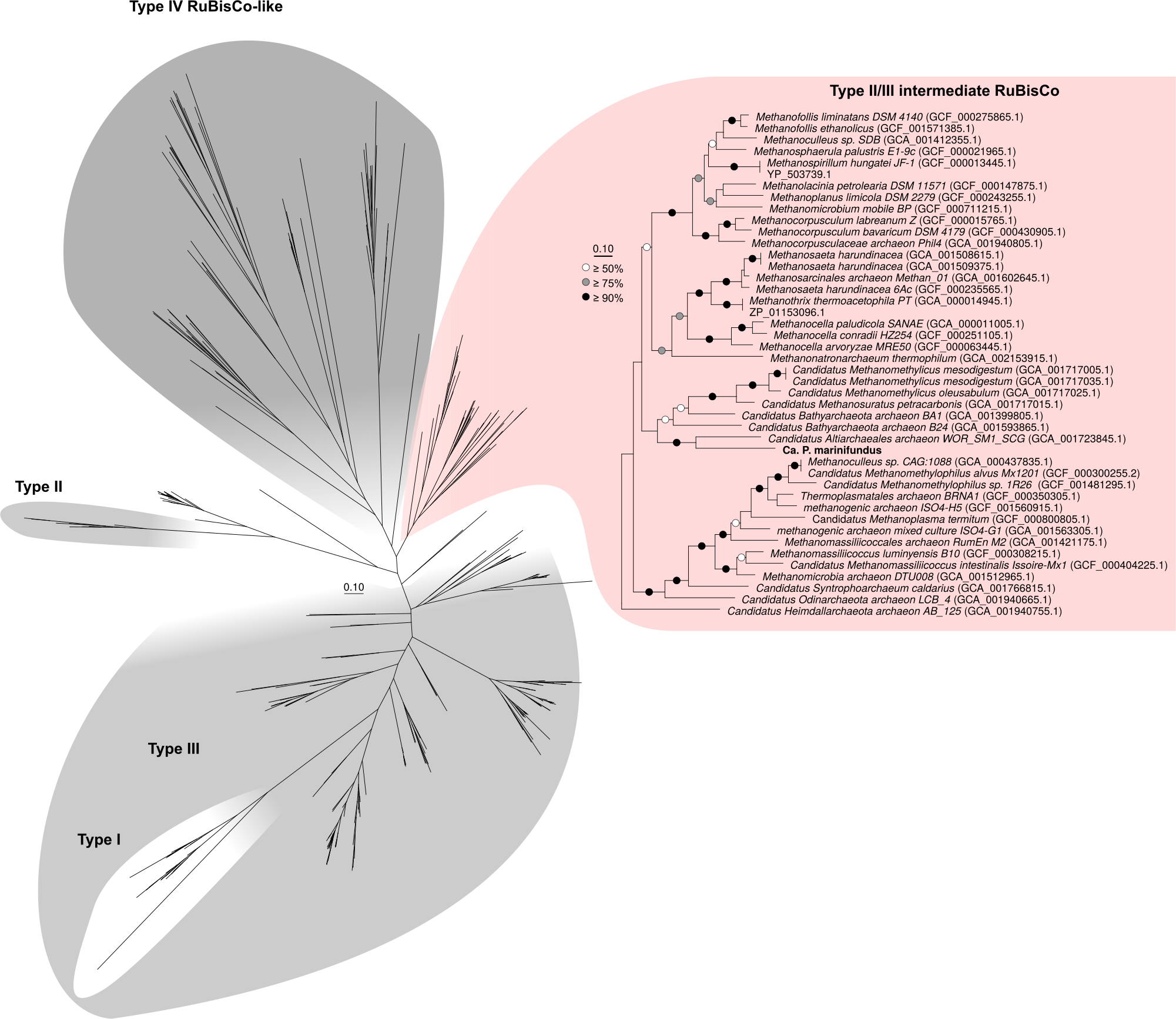
**

**Supplementary Figure 13.** As is Supplementary Figure 12, but constructed using IQ-TREE.


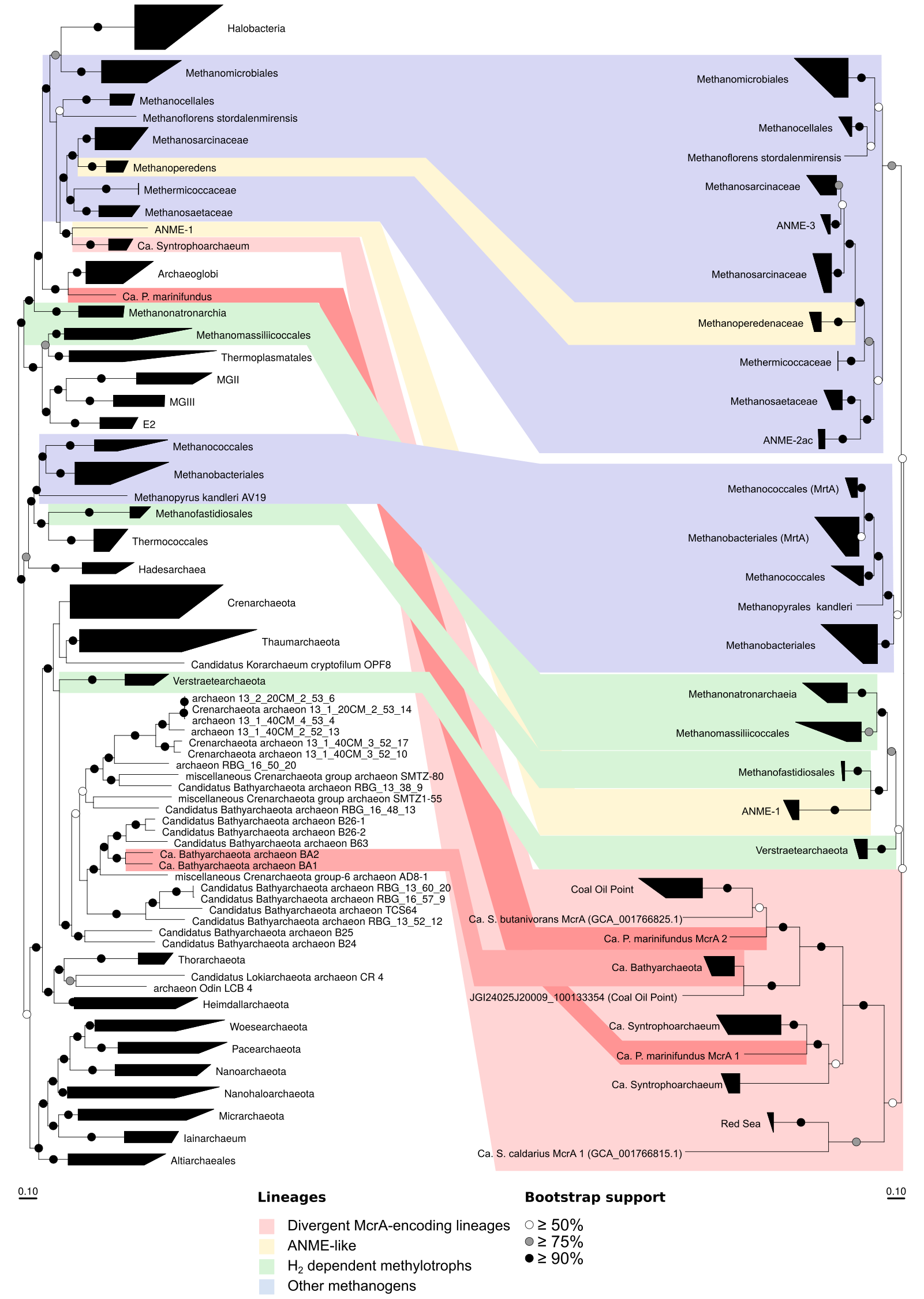


**Supplementary Figure 14.** Comparison of the topology of the McrA gene (**Figure 1A**) tree and archaeal genome tree (**Figure 1B**).

**
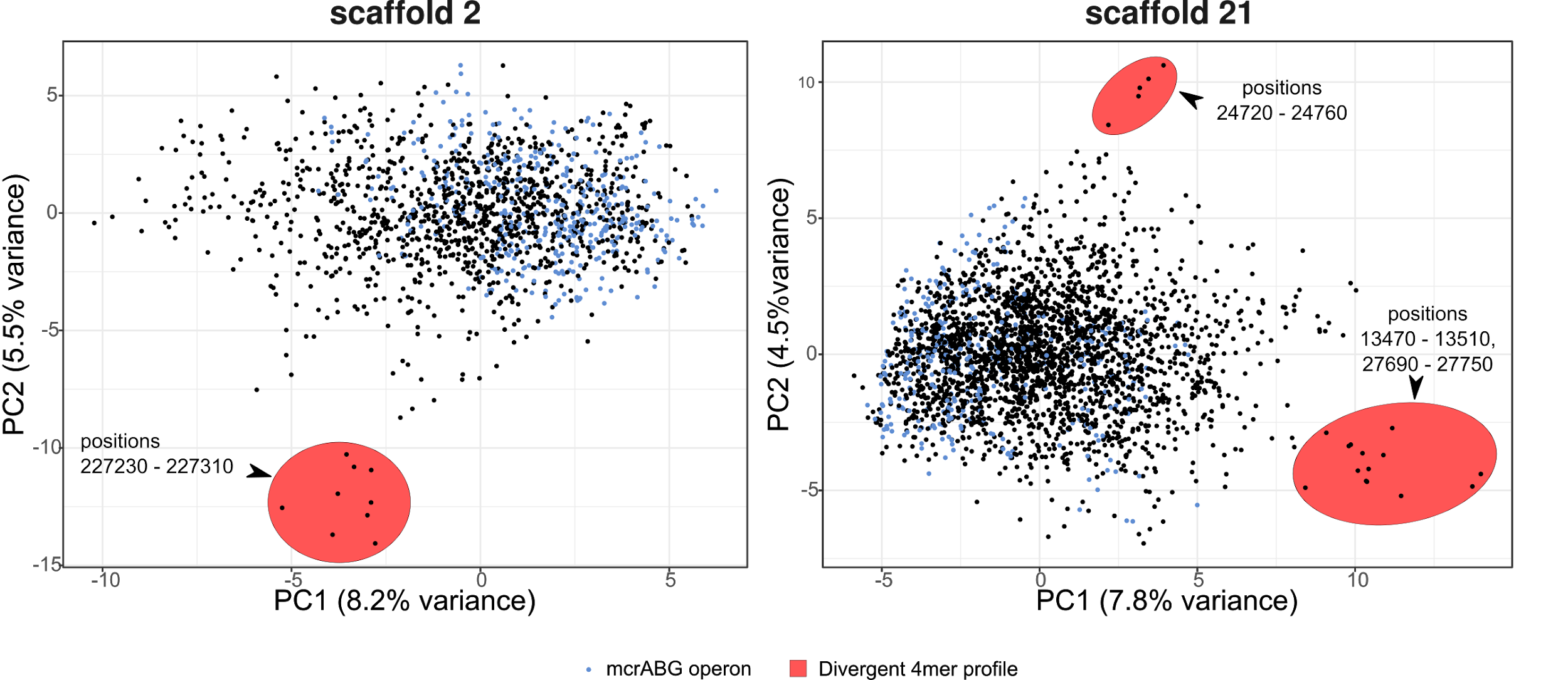
**

**Supplementary Figure 15.** A principal component analysis of the sliding window 4mer profile around McrABG operon. Red circles indicate manually defined regions with divergent 4mer profiles.

**
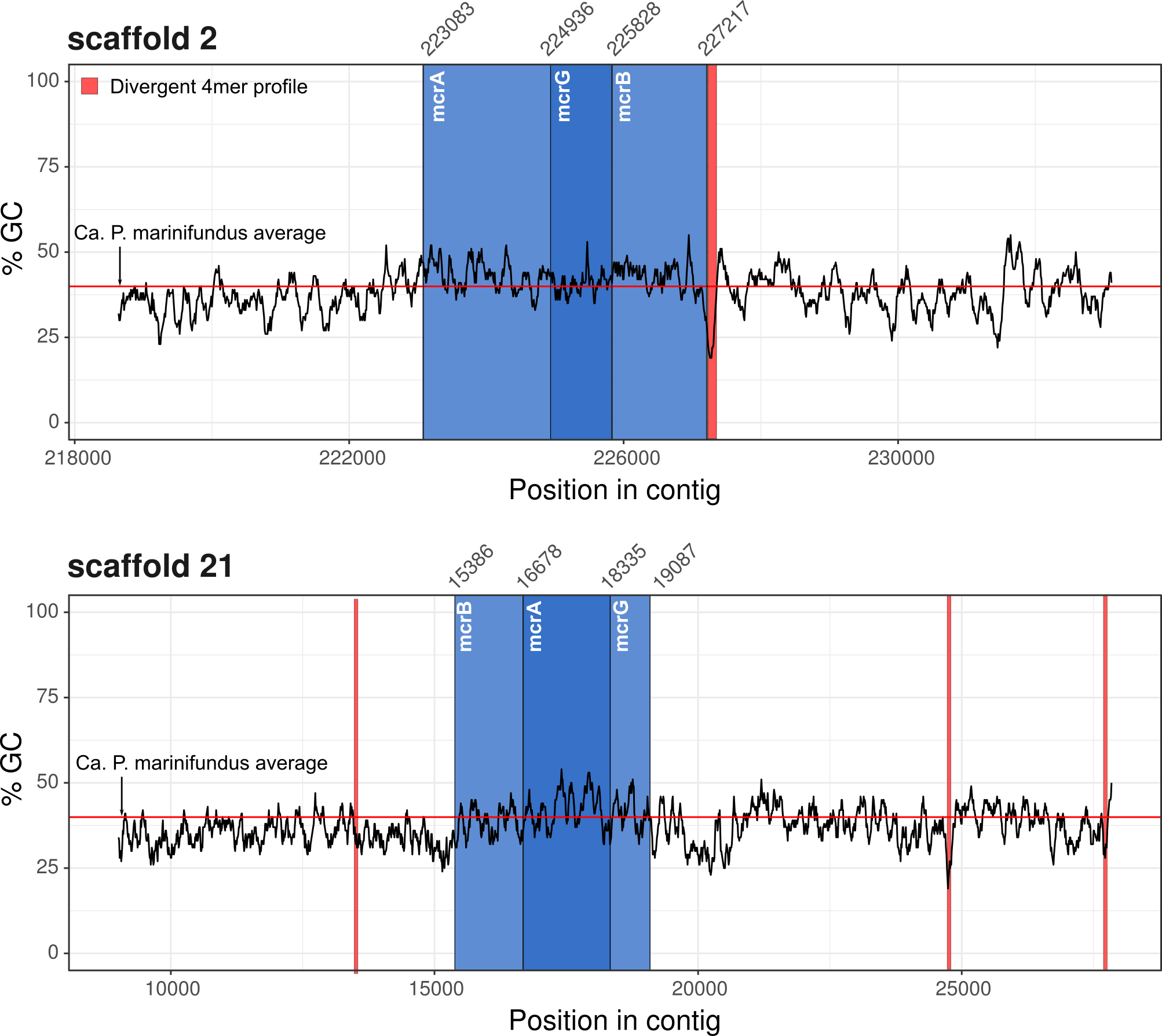
**

**Supplementary Figure 16.** Sliding window %GC content around the *Ca.* P. marinifundus McrABG operons. Red regions correspond to manually defined regions with divergent 4mer profiles in Supplementary Figure 10.

**
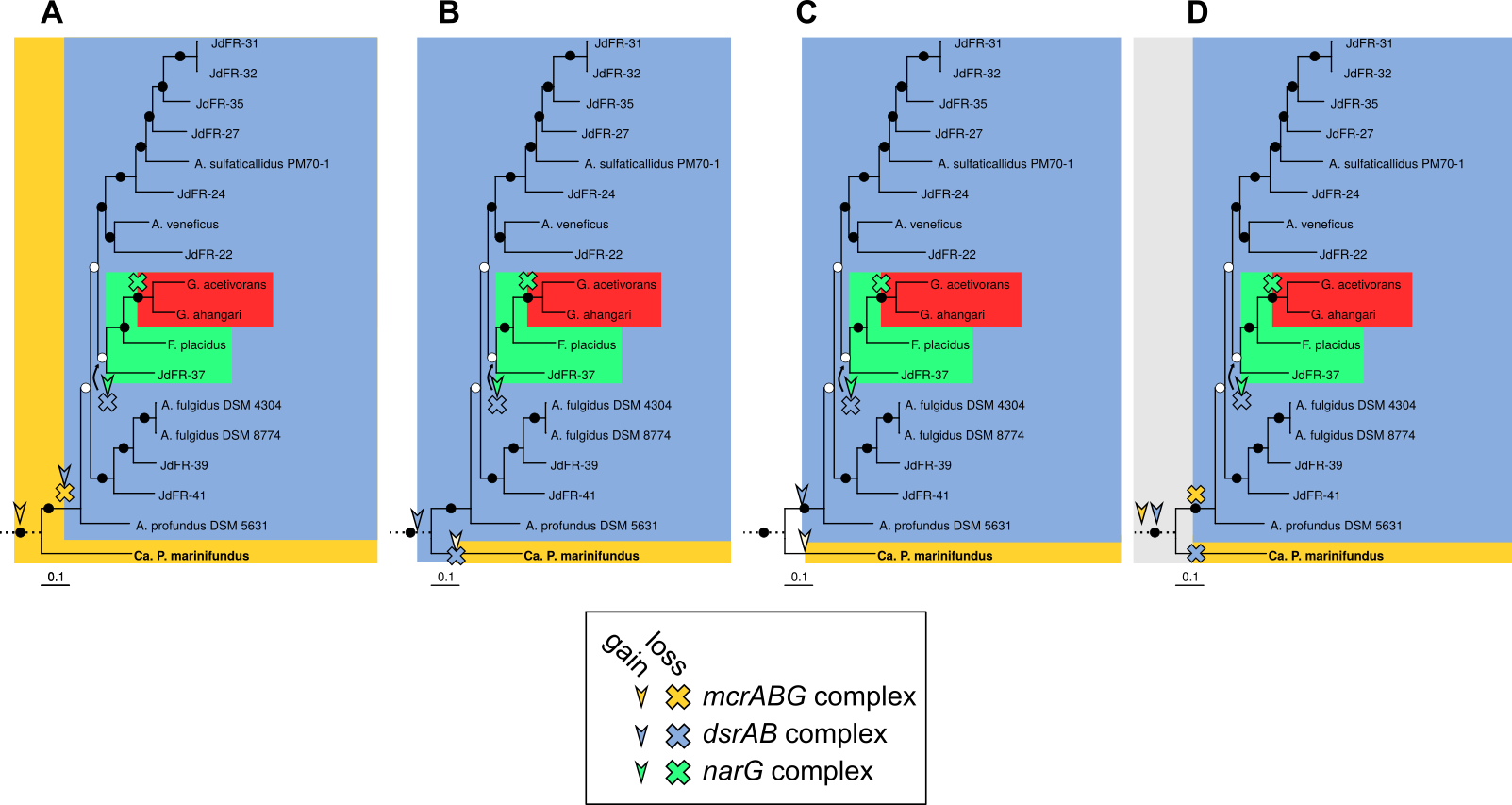
**

**Supplementary Figure 17.** Scenarios that might explain the current distribution of the MCR complex and the *dsrAB* gene among the Archaeoglobi.

# Supplementary Notes

## Supplementary note 1: RefineM analysis to remove contaminant contigs

To remove potential contaminant contigs from the *Ca.* P. marinifundus MAG, RefineM analysis [[2]](https://paperpile.com/c/cShp06/2MIz1) was applied to the contigs using the outliers command. A single, short contig (2748 bp) was identified with a divergent kmer coverage profile (**Supplementary Figure 1.**). Further, 2 of the 4 proteins it encodes shares highest similarity with bacterial homologs. This contig was therefore removed from the *Ca.* P. marinifundus bin.

# **References**

1. [Ermler U, Grabarse W, Shima S, Goubeaud M, Thauer RK. Crystal structure of methyl-coenzyme M reductase: the key enzyme of biological methane formation. *Science* 1997; **278**: 1457–1462.](http://paperpile.com/b/cShp06/gpOn4)

2. [Parks DH, Rinke C, Chuvochina M, Chaumeil P-A, Woodcroft BJ, Evans PN, et al. Recovery of nearly 8,000 metagenome-assembled genomes substantially expands the tree of life. *Nature Microbiology* 2017; **2**: 1533–1542.](http://paperpile.com/b/cShp06/2MIz1)
